# Ferroptosis-related gene signature predicts the clinical outcome in pediatric acute myeloid leukemia patients and refines the 2017 ELN classification system

**DOI:** 10.3389/fmolb.2022.954524

**Published:** 2022-08-11

**Authors:** Yu Tao, Li Wei, Hua You

**Affiliations:** ^1^ Department of Oncology, Affiliated Cancer Hospital & Institute of Guangzhou Medical University, Guangzhou, China; ^2^ NHC Key Laboratory of Birth Defects and Reproductive Health, Chongqing Population and Family Planning Science and Technology Research Institute, Chongqing, China

**Keywords:** ferroptosis, prognostic model, pediatric acute myeloid leukemia, immune infiltration, immune checkpoint (ICP)

## Abstract

**Background:** The prognostic roles of ferroptosis-related mRNAs (FG) and lncRNAs (FL) in pediatric acute myeloid leukemia (P-AML) patients remain unclear.

**Methods:** RNA-seq and clinical data of P-AML patients were downloaded from the TARGET project. Cox and LASSO regression analyses were performed to identify FG, FL, and FGL (combination of FG and FL) prognostic models, and their performances were compared. Tumor microenvironment, functional enrichment, mutation landscape, and anticancer drug sensitivity were analyzed.

**Results:** An FGL model of 22 ferroptosis-related signatures was identified as an independent parameter, and it showed performance better than FG, FL, and four additional public prognostic models. The FGL model divided patients in the discovery cohort (*N* = 145), validation cohort (*N* = 111), combination cohort (*N* = 256), and intermediate-risk group (*N* = 103) defined by the 2017 European LeukemiaNet (ELN) classification system into two groups with distinct survival. The high-risk group was enriched in apoptosis, hypoxia, TNFA signaling *via* NFKB, reactive oxygen species pathway, oxidative phosphorylation, and p53 pathway and associated with low immunity, while patients in the low-risk group may benefit from anti-TIM3 antibodies. In addition, patients within the FGL high-risk group might benefit from treatment using SB505124_1194 and JAK_8517_1739.

**Conclusion:** Our established FGL model may refine and provide a reference for clinical prognosis judgment and immunotherapies for P-AML patients.

## Introduction

Acute myeloid leukemia (AML) encompasses a high heterogeneity hematologic malignancy, characterized by uncontrolled proliferation of myeloid blasts or progranulocytes, leading to suppression of the normal hematopoietic function of bone marrow (Newell and Cook, 2021). Its heterogeneity is related to clinical behavior, morphology, immunophenotyping, germline and somatic genetic abnormalities, and epigenetic anomalies, as well as patient outcomes (Creutzig et al., 2012).

The 2017 European LeukemiaNet (ELN) classification system integrated karyotypic abnormalities and genetic mutations to classify AML patients into three genetic risk groups: favorable, intermediate, and adverse, and subsequently found widespread adoption in clinical practice (Döhner et al., 2017). However, around 50% of AML patients are still classified as the intermediate-risk subgroup, and their survival is highly heterogeneous (Wang et al., 2017), implying the need for integrating additional prognostic factors to improve risk stratification power.

Pediatric acute myeloid leukemia (P-AML), despite constant treatment improvements over the past decades, remains a catastrophic disease with 3-year relapse rates up to 30% and 5-year survival rates below 75% (Unis et al., 2021). Since most of the genetic investigations are based on adult AML patients, distinct molecular genetic landscapes have been mapped out between pediatric and adult AML patients (Bolouri et al., 2018; Marceau-Renaut et al., 2018). It is important to define a better description of the pattern of molecular aberrations in P-AML in order to refine prognostication and develop age-specific therapies in such patients.

Ferroptosis is a new mode of regulated cell death, which is usually accompanied by iron accumulation and lipid peroxidation during the cell death process and is involved in the development of many critical diseases, such as tumors, ischemic tissue damage, kidney injury, neurodegeneration, and blood diseases (Li et al., 2020), especially recent research has shown that ferroptosis-inducing agents and genetic modulators of ferroptosis resulted in a synergistic effect on the promotion of early death of AML cells and increasing the sensitivity of leukemia cells to chemotherapeutic agents (Yu et al., 2015; Birsen et al., 2021). Thus, we reasonably hypothesize that ferroptosis is involved in the pathobiology of AML including pediatric patients. In addition, emerging evidence has proven that high-complexity links between N6-methyladenosine (m6A) and different types of programmed cell death pathways might be closely associated with the initiation, progression, and resistance of cancer (Liu et al., 2022). An intriguing study investigated the mechanisms underlying the oncogenic role of m6A demethylase FTO in AML and found that a bio-imprinted nanoplatform targeting the FTO/m6A pathway can selectively target leukemic stem cells (LSCs) and induce ferroptosis (Cao et al., 2022), implying the clinical potential of targeting “m6A modification–ferroptosis axis” as a treatment strategy against AML.

Currently, a few studies indicated that abnormalities in mRNAs or long non-coding RNAs (lncRNAs) of ferroptosis were closely correlated with cancer patient outcomes (Lelièvre et al., 2020). However, no study has been performed to discover ferroptosis-related prognostic signatures and predict P-AML outcomes.

Here, the main aim of the present study was to explore the prognostic roles of ferroptosis-related mRNAs and lncRNAs for P-AML patients, construct three prognostic models based on the RNA sequencing data of pediatric samples from the TARGET AML cohort (Downing et al., 2012), compare their predictive efficiency among three models to obtain the best one, and investigate the potential benefits of immune therapy. Second, we explored whether the incorporation of the best ferroptosis-related signatures could further improve the prognostic prediction for the intermediate-risk subgroup of the 2017 ELN risk classification system.

## Materials and methods

### Samples and datasets

**FIGURE 1:**
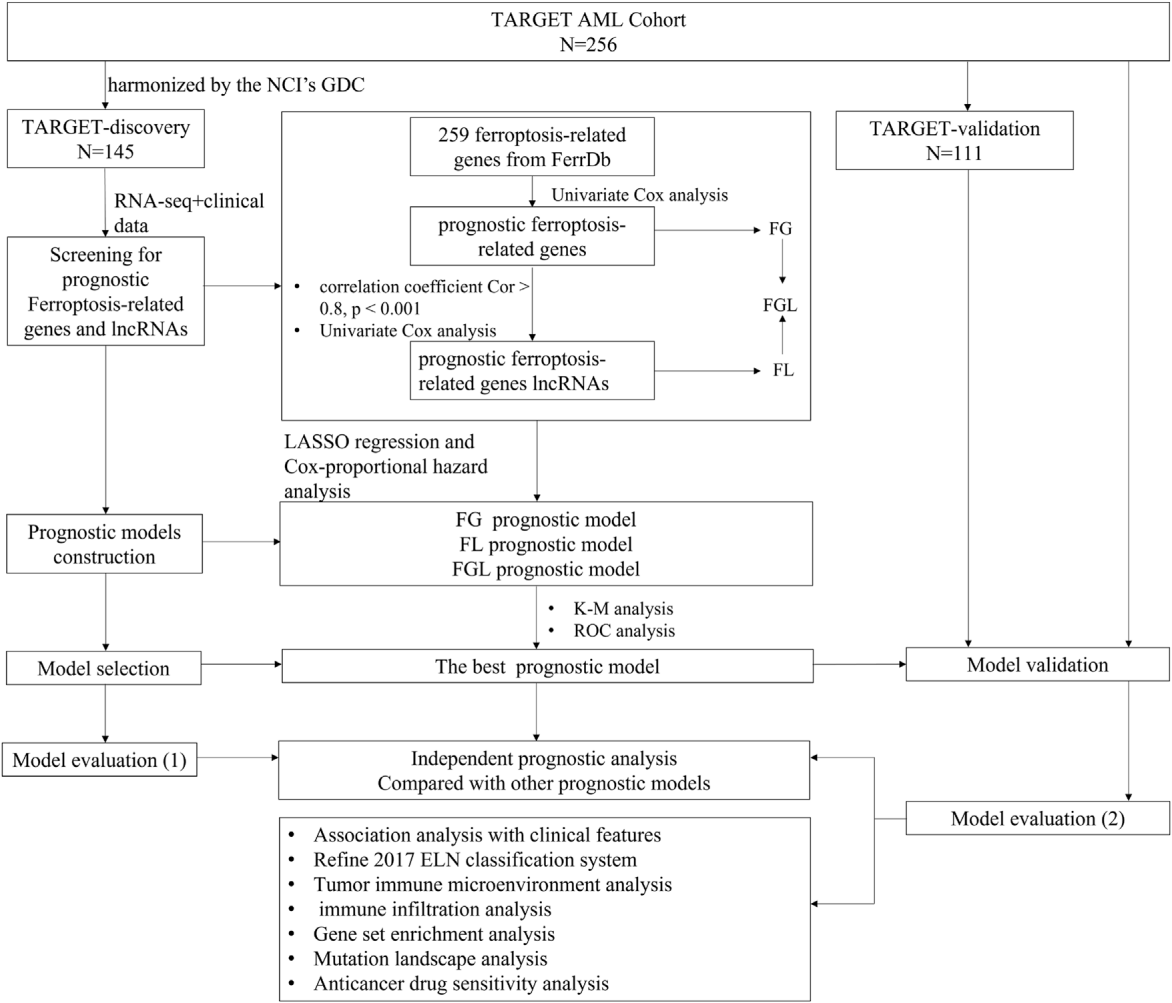
Workflow of this study.

RNA-seq data and clinical information of 256 P-AML samples were collected at the first presentation/diagnosis from the Therapeutically Applicable Research to Generate Effective Treatment (TARGET) AML program and were available from NCI’s data portal (collected on 18/September/2021). Out of these 256 samples, 145 have been harmonized by the NCI’s Genomic Data Commons (GDC) (Zhang et al., 2021). Raw expression counts of these 145 AML patients were extracted TARGET-AML program of GDC (collected on 18/August/2021), as the TARGET-discovery cohort. The remaining 111 cases were used as a validation cohort and raw expression counts were gathered from the (NCI)’s data portal (TARGET-validation cohort). For model validation in the adult AML cohort, RNA-Sequencing data of 151 adult patients with AML and the corresponding clinical information were extracted from the The Cancer Genome Atlas (TCGA)-LAML program of GDC. The “DESeq2” package based on the negative binomial distribution was used to normalize the raw count expression data (Love et al., 2014). Detailed clinical information about the data cohorts is provided in Table 1, and the workflow is briefly depicted in Figure 1.

**TABLE 1:** Summary of P-AML patient clinical information from TARGET-Discovery and validation databases.

	TARGET-GDC	TARGET-111	TAREGT-combined	*p*-value
Patients, *n*	145	111	256	
Gender				0.62
Female	71 (48.97%)	50 (45.05%)	121 (47.27%)	
Male	74 (51.03%)	61 (54.95%)	135 (52.73%)	
Age at diagnosis in days				
Mean ± SD	3,364.50 ± 2,210.76	3,776.92 ± 1980.25	3,543.32 ± 2,119.79	0.12
Median [min-max]	3,438.00 [137.00,8231.00]	4,183.00 [10.00,7442.00]	3,831.00 [10.00,8231.00]	
Cytogenetic abnormality, carriers (%)				
t (8; 21)	21 (14.48%)	23 (20.72%)	44 (17.19%)	0.42
t (6; 9)	1 (0.69%)	2 (1.80%)	3 (1.17%)	0.71
t (3; 5) (q25; q34)	2 (1.38%)	1 (0.90%)	3 (1.17%)	0.92
t (6; 11) (q27; q23)	2 (1.38%)	2 (1.80%)	4 (1.56%)	0.95
t (9; 11) (p22; q23)	13 (8.97%)	5 (4.50%)	18 (7.03%)	0.37
t (10; 11) (p11.2; q23)	4 (2.76%)	2 (1.80%)	6 (2.34%)	0.86
t (11:19) (q23:p13.1)	5 (3.45%)	0	5 (1.95%)	0.14
inv (16)	28 (19.31%)	14 (12.61%)	42 (16.41%)	0.34
del5q	1 (0.69%)	0	1 (0.39%)	0.67
del7q	4 (2.76%)	5 (4.50%)	9 (3.52%)	0.75
del9q	5 (3.45%)	5 (4.50%)	10 (3.91%)	0.90
trisomy 8	9 (6.21%)	9 (8.11%)	18 (7.03%)	0.83
trisomy 21	4 (2.76%)	1 (0.90%)	5 (1.95%)	0.21
Minus Y	6 (4.14%)	5 (4.50%)	11 (4.30%)	0.97
Minus X	6 (4.14%)	4 (3.60%)	10 (3.91%)	0.96
FLT3_ITD_positive	11 (7.59%)	29 (26.13%)	40 (15.63%)	**<0.001**
AML with biallelic mutations of CEBPA	7 (4.86%)	9 (8.33%)	16 (6.25%)	0.41
AML with mutated WT1	8 (5.67%)	9 (8.33%)	17 (6.64%)	0.83
AML with mutated NPM1	5 (3.45%)	13 (12.26%)	18 (7.03%)	0.07
Median WBC count (range), 3×10^9^/L	45.30 [1.30,519.00]	42.80 [0.90,432.00]	44.75 [0.90,519.00]	0.36
Median percentage of BM blasts (range)	74.80 [14.00,100.00]	72.00 [25.00,98.00]	73.00 [14.00,100.00]	0.41
Median percentage of PB (range)	61.00 [0.0e+0,97.00]	61.00 [0.0e+0,97.00]	61.00 [0.0e+0,97.00]	0.28
2017 ELN classification system, *n* (%)				**<0.001**
Favorable	60 (41.38%)	52 (46.85%)	112 (43.75%)	
Intermediate	69 (47.59%)	34 (30.63%)	103 (40.23%)	
Adverse	8 (5.52%)	20 (18.02%)	28 (10.94%)	
Unknown	8 (5.52%)	5 (4.50%)	13 (5.08%)	
FAB subtype				0.15
M0	3 (2.07%)		3 (1.17%)	
M1	17 (11.72%)	16 (14.41%)	33 (12.89%)	
M2	35 (24.14%)	31 (27.93%)	66 (25.78%)	
M4	36 (24.83%)	26 (23.42%)	62 (24.22%)	
M5	30 (20.69%)	19 (17.12%)	49 (19.14%)	
M6	2 (1.38%)	1 (0.90%)	3 (1.17%)	
M7	7 (4.83%)	6 (5.41%)	7 (2.73%)	
NOS	8 (5.52%)	12 (10.81%)	14 (5.47%)	
Unknown	7 (4.83%)	16 (14.41%)	19 (7.42%)	
CNS disease				0.70
No	135 (93.10%)	101 (90.99%)	236 (92.19%)	
Yes	10 (6.90%)	10 (9.01%)	20 (7.81%)	
Chloroma				**0.03**
No	134 (93.06%)	92 (82.88%)	226 (88.28%)	
Yes	10 (6.94%)	19 (17.12%)	29 (11.33%)	
Unknown	1 (0.69%)		1 (0.39%)	
MRD at the end of course 1				0.14
No	83 (75.45%)	52 (46.85%)	135 (52.73%)	
Yes	27 (24.55%)	20 (18.02%)	47 (18.36%)	
Unknown	35 (13.67%)	39 (35.13%)	74 (28.91%)	
HSCT, *n*				0.06
No	131 (90.34%)	89 (80.18%)	220 (85.94%)	
Yes	13 (8.97%)	19 (17.11%)	32 (12.50%)	
Yes	1 (0.69%)	3 (2.71%)	4 (1.56%)	
Event-free survival time in days				
Mean ± SD	954.70 ± 972.36	1,058.67 ± 1,022.27	999.78 ± 993.67	0.41
Median [min-max]	458.00 [77.00,3630.00]	506.00 [2.00,4037.00]	461.00 [2.00,4037.00]	
Overall Survival Time in days				
Mean ± SD	1,577.18 ± 1,064.78	1,446.80 ± 1,008.87	1,520.65 ± 1,040.91	0.32
Median [min-max]	1,464.00 [112.00,4022.00]	1,532.00 [2.00,4037.00]	1,523.50 [2.00,4037.00]	
Vital Status				0.24
Alive	77 (53.10%)	68 (61.26%)	145 (56.64%)	
Dead	68 (46.90%)	43 (38.74%)	111 (43.36%)	

Notes: WBC, white blood cell; BM, bone marrow; PB, peripheral blast; ELN, European LeukemiaNet; FAB, French–American–British; NOS, not otherwise specified; CNS, central nervous system; MRD, measurable residual disease; HSCT, hematopoietic stem-cell transplantation; SD, standard deviation.

### Ferroptosis-related mRNAs and lncRNAs

We obtained 259 ferroptosis-related genes from the FerrDb database (http://www.zhounan.org/ferrdb, collected on 16/November/2021), the first manually curated resource for regulators and markers of ferroptosis, which was released in January 2020 (Zhou and Bao, 2020) (Supplementary Table S1). LncRNAs were extracted by the GENCODE v20 annotation (http://www.gencodegenes.org) (Derrien et al., 2012). Ferroptosis-related lncRNAs co-expressed with ferroptosis-related genes were identified according to Pearson’s correlation analysis by the correlation test function of R (correlation coefficient Cor >0.8, *p* < 0.001).

### Prognostic model construction and validation

Univariate Cox regression was applied to screen prognosis-related mRNAs and lncRNAs in the discovery cohort, using *p* < 0.05 as the cutoff. Then, model-making procedures were conducted for three lists of prognostic ferroptosis-related signatures separately, ferroptosis-related mRNA genes (FG), ferroptosis-related lncRNAs (FL), and ferroptosis-related mRNA genes combined with ferroptosis-related lncRNAs (FGL), and consisted of the following steps. Step 1, the least absolute shrinkage and selection operator (LASSO) Cox regression analysis was conducted on the prognosis-related signatures, and the optimal penalty parameter “λ” was selected by the 10-fold cross-validation method. Step 2, the prognostic risk score formula was established as follows: risk score = expression of gene 1×*Coef*1+expression of gene 2 × *Coef* 2+ ⋯ +expression of gene *n* × *Coefn*. *Coef* indicates the regression coefficients of each signature selected from the LASSO regression analysis. According to the risk model, samples in the discovery cohort were given a risk score and then divided into high- and low-risk groups using the median score as the cutoff. The survival curves of the patients in the high- and low-risk groups were drawn with the R-package “Survival,” and the survival time of the two groups was compared by log-rank test. The receiver operating characteristic (ROC) curves were drawn using the R package “survival ROC” for validation of the risk model and the AUC values of 1-, 3-, and 5-year survival were calculated. The best prognostic model identified with prognostic significance was selected for further analysis and the same algorithm was performed in the TARGET-validation cohort, TARGET-combined cohort, and TCGA-LAML cohort (151 adult patients), with the same coefficients derived for the discovery dataset.

### Tumor immune microenvironment analysis

Tumor microenvironment, immune, and stroma scores were calculated based on the gene expression data using ESTIMATE (Yoshihara et al., 2013) and xCell (https://xcell.ucsf.edu) tool (Aran et al., 2017). Estimating the proportion of immune and cancer cells (EPIC) was applied to estimate the infiltration ratio of eight types of immune cells (Racle et al., 2017). Furthermore, based on the review of relevant literature (Gong et al., 2018; Rowshanravan et al., 2018; Qin et al., 2019; Wang et al., 2020), we explored the difference in the expression of 10 potential immune checkpoint genes between groups. Detailed information is provided in Supplementary Note S1.

### Functional and pathway enrichment analysis

Gene set enrichment analysis (GSEA) on the 50 hallmark gene sets was used to identify the potential molecular mechanisms or potential functional pathways that involve the prognostic model (Liberzon et al., 2015). Significant gene sets were based on the following parameters: normalized enrichment score (NES) | >1, nominal *p*-value < 0.05, and false discovery rate (FDR) *q*-value < 0.05. Furthermore, 19 m6A-related genes and regulators based on Juan Xu’s research (Li et al., 2019) were further screened and investigated in high- and low-risk groups.

### Sensitivity analysis of common chemotherapeutic drugs

To evaluate the potential of FGL models in clinical practice for P-AML treatment, the half-maximal inhibitory concentration (IC_50_) of commonly administered chemotherapeutic drugs in the TARGET-combined cohort was calculated using the algorithm R package “oncoPredict” (Maeser et al., 2021). The algorithm predicts the clinical drug response value (IC_50_) in patients based on gene expression data in tumor, which is derived from the ridge regression model based on drug sensitivity data from the Genomics of Drug Sensitivity in *Cancer* (GDSC) database.

### Statistical analysis

Kaplan–Meier curves were plotted to estimate overall survival (OS) time and event-free survival (EFS) time, and the data were statistically compared with the log-rank test. Univariate and multivariate analyses were performed by the Cox proportional hazard model. The ROC curve was established, and the area under the curve (AUC) was calculated to determine the predictive value of the prognostic model. Five-fold cross-validation was applied for model evaluation (Supplementary Note S2). All statistical analyses were carried out using R (3.5.2) software and *p* < 0.05 was taken as being statistically significant. The Wilcoxon test was used to compare the immune scores, immune infiltrate, and expression of genes between groups.

## Results

### Prognostic ferroptosis-related signatures are identified

Normalized gene expression data of the TARGET-discovery cohort included 244 ferroptosis-related genes. Univariate Cox regression analysis revealed 39 ferroptosis-related genes with significant prognostic value for P-AML (*p* < 0.05) (Supplementary Table S2). By ferroptosis-related lncRNA co-expression analysis and univariate Cox regression analysis, we identified 8 prognostic ferroptosis-related lncRNAs out of 2,734 co-expressed lncRNAs (Supplementary Table S2). These 39 ferroptosis-related genes and 8 lncRNAs and merged lists were served as candidate lists for LASSO Cox regression analysis, respectively. After optimal parameter (lambda) selection in the LASSO regression, three prognostic ferroptosis-related signature models with 20 FG components, 6 FL components, and 22 FGL components were built, respectively (Supplementary Figure S1; Supplementary Table S3). The expression level of each gene and LASSO regression coefficient (*β*) were integrated to calculate the FG, FL, and FGL risk score for each patient (Supplementary Table S4).

### Prognostic ferroptosis-related models are established and optimized

**FIGURE 2:**
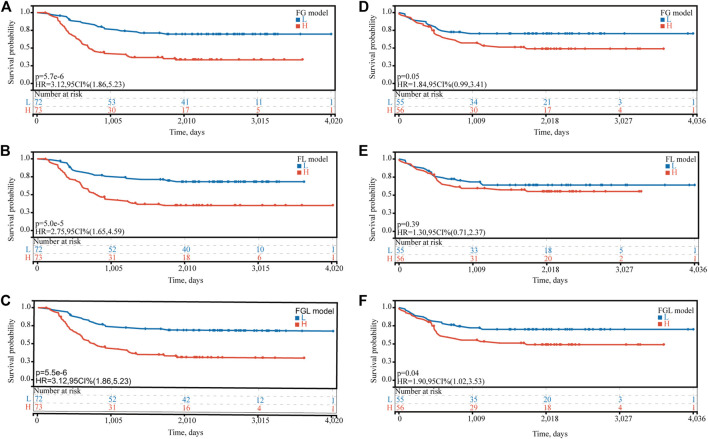
Predictive ability of the FG, FL, and FGL prognostic model for the prognosis of P-AML. Based on the median risk score of **(A**,**D)** FG, **(B**,**E)** FL, and **(C**,**F)** FGL models, patients were divided into the high-risk group and the low-risk group. **(A,B,C)** Kaplan–Meier curve of the high-risk and low-risk groups of the TARGET-discovery cohort. **(D,F)** Kaplan–Meier curve of the high-risk and low-risk groups of the TARGET-validation cohort.

**FIGURE 3:**
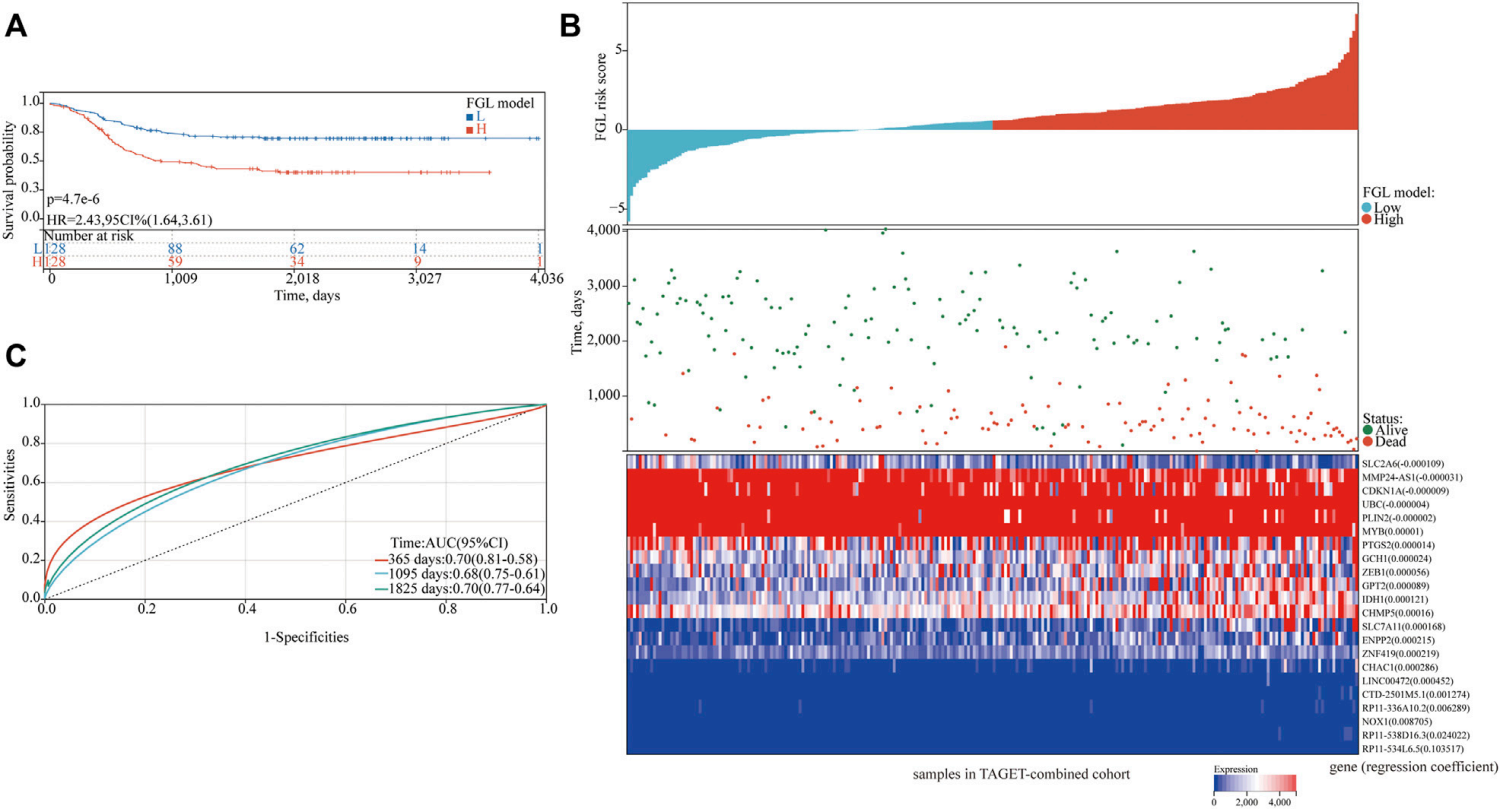
Characteristics of the patients in the TARGET-combined cohort based on the constructed FGL model. **(A)** Kaplan–Meier curve of the high-risk and low-risk groups of the combination set showed differences in survival rate. **(B)** Distribution of risk score, survival status of each patient, and heatmap expression of 22 ferroptosis-related signatures in high-risk and low-risk groups were presented. **(C)** Receiver operating characteristic (ROC) curves of 1-year, 3-year, and 5-year overall survival for P-AML patients of the TARGET-combined cohort based on the FGL prognostic model.

In the TARGET-discovery cohort, patients were stratified into high- and low-risk groups using the median risk score as the cutoff value. Statistical differences in overall-survival probability have been identified for the patient groups stratified by the cutoff point of FG, FL, and FGL risk scores, and the survival rate of P-AML patients in the low-risk group of the three models was significantly higher than that in the high-risk group (all *p* < 0.05) (Figures 2A–C). However, only the FGL model was validated in both the TARGET-validation cohort (*p* = 0.04, Figures 2D‐F) and the TARGET-combined cohort (*p* < 0.001, Figure 3A), with significant differences in the survival rate between the two risk groups. The same results have been found for the event-free survival rate (EFS) (Supplementary Figure S2). Thus, the FGL risk score was selected for all subsequent analyses. The distribution and status of OS of the TARGET-combined cohort were then analyzed by ranking the risk scores (Figure 3B). The results showed that patients with higher FGL risk scores had a worse prognosis. Expression profiles of the 22 ferroptosis-related signatures are listed in the heatmap of Figure 3B. The AUC corresponding to 1-, 3- and 5-year OS in the TARGET-combined cohort were 0.70, 0.68, and 0.70, respectively, indicating that the predictive efficiency of the model was good (Figure 3C).

### Increasing FGL risk score is an independent predictor for poorer OS

**FIGURE 4:**
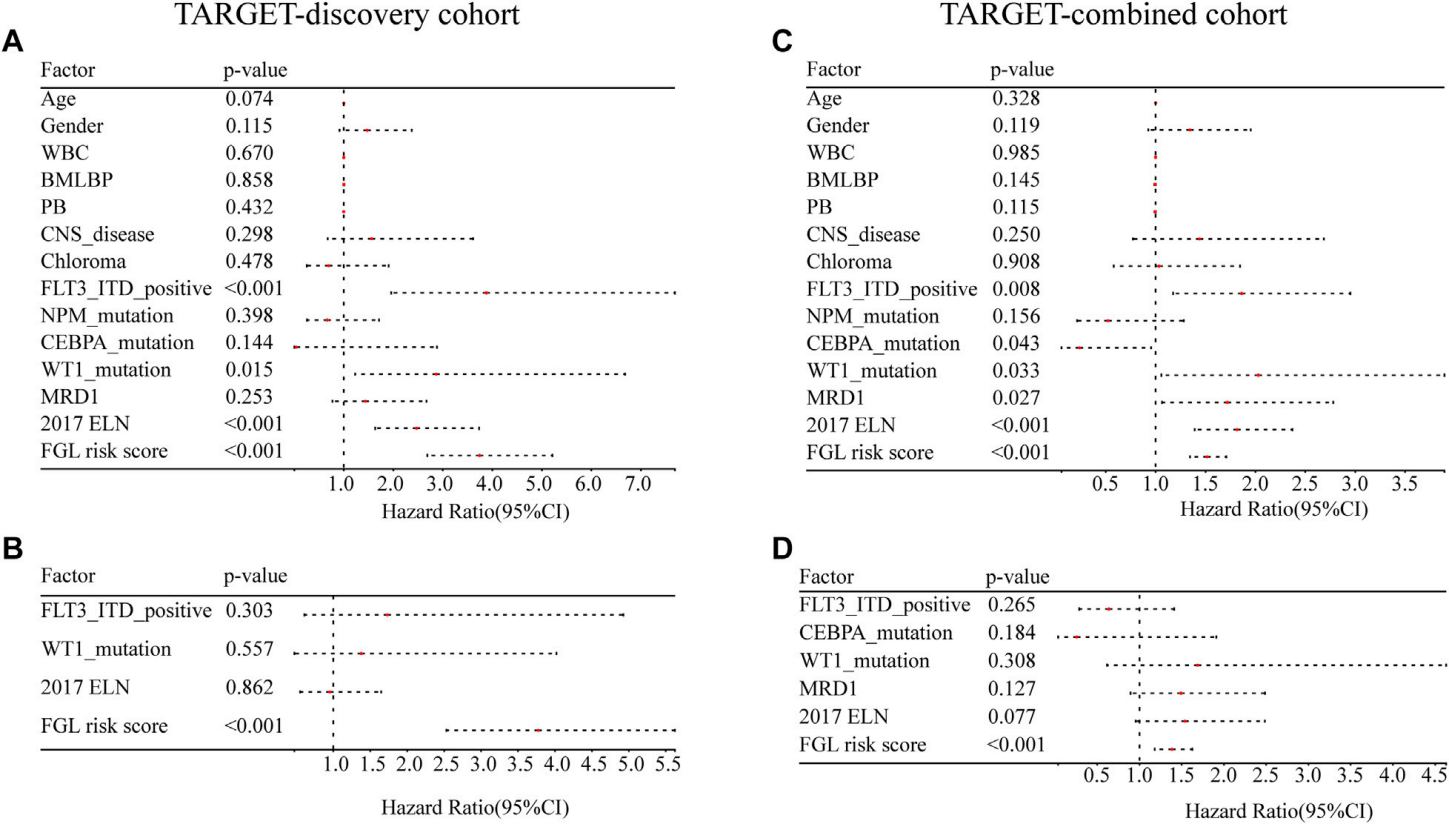
Evaluation of the FGL model in the TARGET-discovery and -combined cohort. Independent prognostic effects of the risk score model were assessed by **(A,C)** univariate Cox regression analysis and **(B,D)** multivariate Cox regression analysis.

**FIGURE 5:**
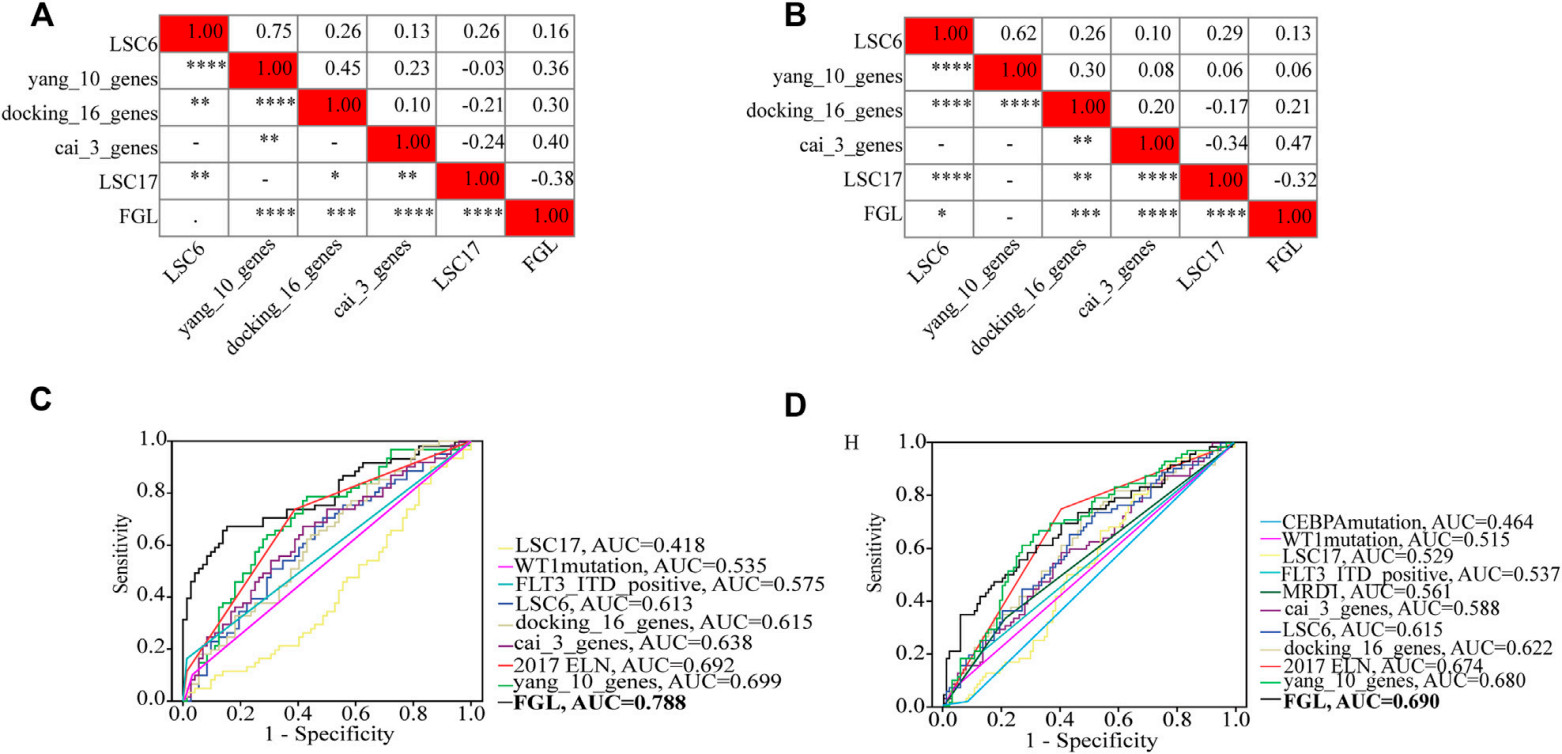
Heatmap showing the correlations between FGL risk score and other prognostic models identified or validated in the TARGET cohort **(A,B)**. FGL prognostic model had the best AUC value in TARGET-discovery cohort **(C)** and -combined cohort **(D)**.

FLT3_ITD_positive, WT1 mutation, 2017 ELN classification system, and FGL risk score were considered significant risk parameters in the univariate analysis (*p* < 0.05) (Figure 4A), and further multivariate Cox analysis indicated that the FGL risk score was the only independent risk parameter in the discovery cohort (HR = 3.772, 95% CI = 2.529–5.625) (Figure 4B). In the TARGET-combined cohort, the results indicated that the higher FGL risk score was also the only independent poor prognosticator for OS (HR = 1.515, 95% CI = 1.344–1.708) (Figures 4C,D). Several prognostic models have been established or validated in the TARGET cohort recently: LSC17 (Duployez et al., 2019), LSC6 (Elsayed et al., 2020), yang_10_genes (Yang et al., 2020), docking_16_genes (Docking et al., 2021), and cai_3_genes (Cai et al., 2021). Risk scores were further calculated based on the coefficient defined in these studies and used further for correlation analysis with FGL risk score. FGL risk score was negatively correlated with LSC17 risk score and positively correlated with the docking_16_genes model and cai_3_genes model (Figures 5A,B). FGL risk model demonstrated the best predictive performance compared with previous prognostic models, 2017 ELN classification system, and other prognostic molecular characteristics in TARGET-discovery and -combined cohort, respectively (Figures 5C,D). The five-fold cross-validation method was applied to give a robust estimation of the performance of the FGL model. As shown in Supplementary Table S5, in the testing stage, the AUC of the FGL model ranged from 0.693 to 0.741 in the TARGET-discovery cohort and 0.693 to 0.741 in the combined cohort. Collectively, the risk score calculated according to the 22 ferroptosis-related signatures could serve as an independent and stable prognostic parameter for P-AML patients. However, we further explored the predictive ability of the FGL prognostic model for prognosis in adult AML patients. OS was not significantly different in the high-risk and low-risk groups of the TCGA-LAML cohort (*p* = 0.37, Supplementary Figure S3).

### Evaluation of the relationship between clinicopathological and molecular characteristics and the ferroptosis-related signature

**FIGURE 6:**
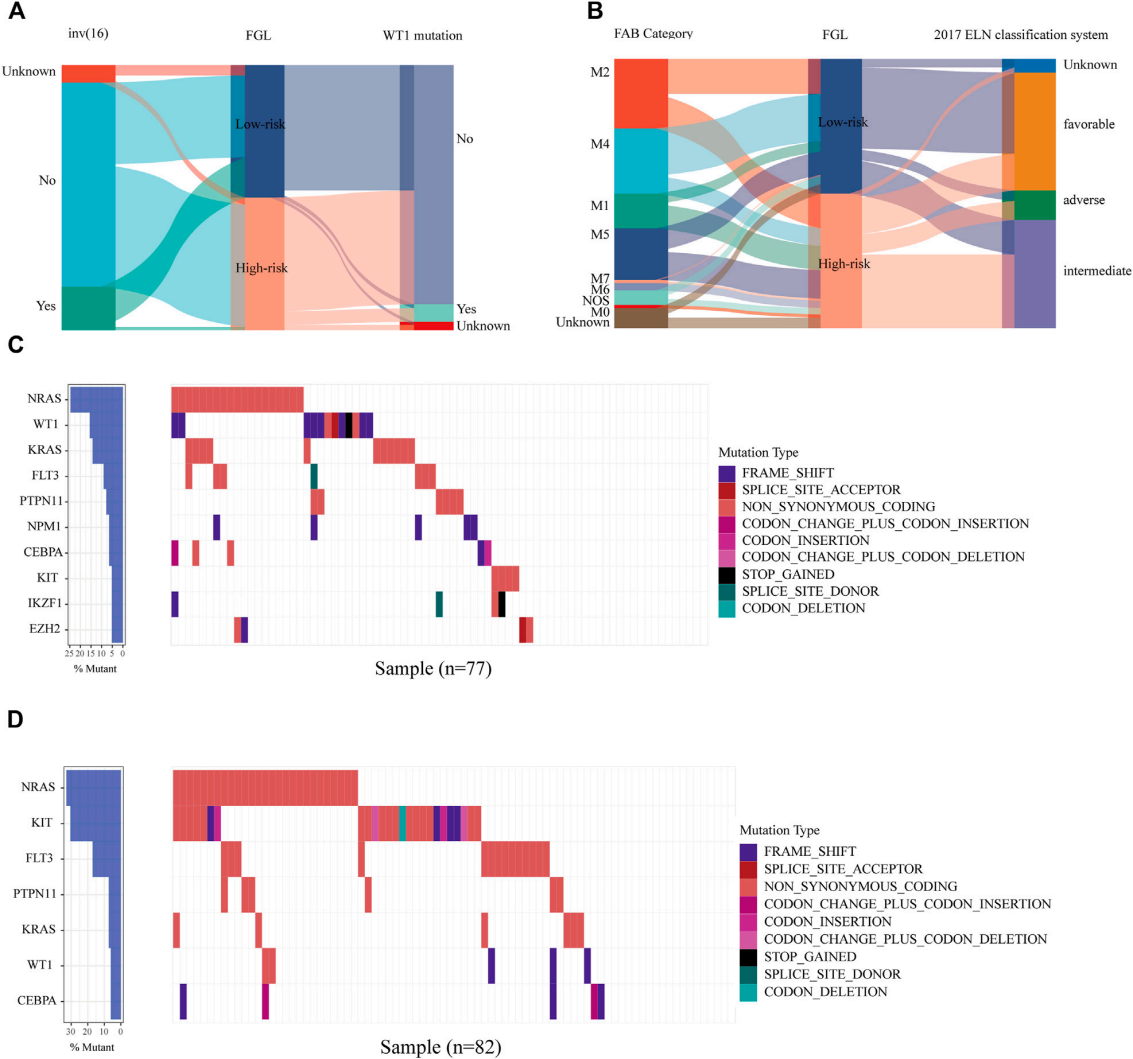
Relationship between clinicopathological and molecular characteristics and the ferroptosis-related signature. Sankey diagram describing the relative flow of subgroups of **(A)** inv (16) mutation, WT1 mutation, **(B)** FAB category, and 2017 ELN classification system according to the FGL risk group. Comparison of the mutation landscape between groups with **(C)** high and **(D)** low FGL risk scores.

We next investigated the clinical, molecular, and immune features of the low- and high-risk groups in the TARGET-combined cohort, considering the sample size. The results identified a significant difference between the two groups with respect to the distribution of inv (16) mutation, WT1 mutation, 2017 ELN classification system, and FAB subtype (all *p* < 0.05, Table 2). Of the 42 patients with inv (16), 7.14% (3/42) were in the high-risk group and 92.86% (39/42) were in the low-risk group (*p* < 0.001) (Figure 6A); WT1 mutation occurs in 6.85% (17/256) of AML patients and 13 of them were in the high-risk group (*p* = 0.04) (Figure 6A). Consistent with expectations, the number of people identified as adverse-risk by the 2017 ELN classification system was significantly higher in the high-risk group than that of the low-risk group (14.06 vs. 7.81%), while the number of people identified as favorable-risk was on the contrary (27.34 vs. 60.16%). The morphological subtypes in order of frequency among P-AML cases were M2 (66/256, 25.78%), M4 (62/256, 24.22%), M5 (49/256, 19.14%), M1 (33/256, 12.89%), M7 (7/256, 2.73%), and M0 and M6 (3/256 and 1.17%). No M3 case was identified in the present study. M4 subtype was more common in the low-risk group than in the high-risk group (35.16 vs. 13.28%) (Figure 6B). The mutation landscapes in the FGL high- and low-risk groups showed top mutated genes with a frequency above 5%, and NARS mutation was the predominant alteration in both the high- and low-risk groups. The second highest mutation rate (30.49%) was found for KIT in the high-risk group, while the mutation rate is 5.19% in the low-risk group (*p* < 0.001) (Figures 6C,D).

**TABLE 2:** Clinicopathological Characteristics for high and low FGL risk score subgroups.

	High-risk group	Low-risk group	TAREGT-combined	*p*-value
Patients, *n*	128	128	256	
Gender				0.620
Female	63 (49.22%)	58 (45.31%)	121 (47.27%)	
Male	65 (50.78%)	70 (54.69%)	135 (52.73%)	
Age at diagnosis in days				0.882
Mean ± SD	3,563.05 ± 2094.95	3,523.59 ± 2,152.39	3,543.32 ± 2,119.79	
Median [min-max]	3,810.50 [10.00,7442.00]	3,871.50 [113.00,8231.00]	3,831.00 [10.00,8231.00]	
Cytogenetic abnormality				
t (8; 21) carriers (%)	17 (13.28%)	27 (21.09%)	44 (17.19%)	0.16
t (6; 9)	3 (2.34%)	0	3 (1.17%)	0.17
t (3; 5) (q25; q34)	3 (2.34%)	0	3 (1.17%)	0.17
t (6; 11) (q27; q23)	3 (2.34%)	1 (0.78%)	4 (1.56%)	0.46
t (9; 11) (p22; q23)	10 (7.81%)	8 (6.25%)	18 (7.03%)	0.70
t (10; 11) (p11.2; q23)	4 (3.13%)	2 (1.56%)	6 (2.34%)	0.55
t (11:19) (q23:p13.1)	4 (3.13%)	1 (0.78%)	5 (1.95%)	0.31
inv (16)	3 (2.34%)	39 (30.47%)	42 (16.41%)	**<0.001**
del5q	1 (0.78%)	0	1 (0.39%)	0.46
del7q	3 (2.34%)	6 (4.69%)	9 (3.52%)	0.43
del9q	4 (3.13%)	6 (4.69%)	10 (3.91%)	0.59
trisomy 8	12 (9.38%)	6 (4.69%)	18 (7.03%)	0.28
trisomy 21	5 (3.91%)	0	5 (1.95%)	0.06
Minus Y	3 (2.34%)	8 (6.25%)	11 (4.30%)	0.21
Minus X	5 (3.91%)	5 (3.91%)	10 (3.91%)	0.75
FLT3_ITD_positive	25 (19.53%)	15 (11.72%)	40 (15.63%)	0.12
AML with biallelic mutations of CEBPA	8 (6.40%)	8 (6.25%)	16 (6.35%)	1.00
AML with mutated WT1	13 (10.57%)	4 (3.20%)	17 (6.85%)	**0.04**
AML with mutated NPM1	11 (9.02%)	7 (5.65%)	18 (7.32%)	0.44
Median WBC count (range), 3×10^9^/L	30.25 [0.90,519.00]	56.45 [1.60,405.50]	44.75 [0.90,519.00]	0.24
Median percentage of BM blasts (range)	72.00 [14.00,100.00]	74.30 [21.00,100.00]	73.00 [14.00,100.00]	0.38
Median percentage of PB (range)	61.00 [0.0e+0,97.00]	61.00 [0.0e+0,97.00]	61.00 [0.0e+0,97.00]	0.06
2017 ELN classification system, *n* (%)				**<0.001**
Favorable	35 (27.34%)	77 (60.16%)	112 (43.75%)	
Intermediate	70 (54.69%)	33 (25.78%)	103 (40.23%)	
Adverse	18 (14.06%)	10 (7.81%)	28 (10.94%)	
Unknown	5 (3.91%)	8 (6.25%)	13 (5.08%)	
FAB subtype				**<0.001**
M0	3 (2.34%)	0	3 (1.17%)	
M1	23 (17.97%)	10 (7.81%)	33 (12.89%)	
M2	33 (25.78%)	33 (25.78%)	66 (25.78%)	
M4	17 (13.28%)	45 (35.16%)	62 (24.22%)	
M5	27 (21.09%)	22 (17.19%)	49 (19.14%)	
M6	2 (1.56%)	1 (0.78%)	3 (1.17%)	
M7	7 (5.47%)	0	7 (2.73%)	
NOS	6 (4.69%)	8 (6.25%)	14 (5.47%)	
Unknown	10 (7.81%)	9 (7.03%)	19 (7.42%)	
CNS disease				0.10
No	122 (95.31%)	114 (89.06%)	236 (92.19%)	
Yes	6 (4.69%)	14 (10.94%)	20 (7.81%)	
Chloroma				0.71
No	114 (89.76%)	112 (87.50%)	226 (88.63%)	
Yes	13 (10.24%)	16 (12.50%)	29 (11.37%)	
Unknown			1 (0.39%)	
MRD				0.11
No	63 (49.22%)	72 (56.25%)	135 (52.73%)	
Yes	30 (23.44%)	17 (13.28%)	47 (18.36%)	
Unknown	35 (27.34%)	39 (30.47%)	74 (28.91%)	
HSCT, *n*				0.11
No	110 (85.94%)	110 (85.94%)	220 (85.94%)	
Yes	14 (10.94%)	18 (14.06%)	32 (12.50%)	
Unknown	4 (3.13%)	0	4 (1.56%)	
Event-free survival time in days				**0.001**
Mean ± SD	800.63 ± 895.12	1,198.93 ± 1,049.46	999.78 ± 993.67	
Median [min-max]	379.50 [2.00,3632.00]	653.00 [80.00,4037.00]	461.00 [2.00,4037.00]	
Overall survival time in days				**<0.001**
Mean ± SD	1,228.48 ± 962.09	1812.81 ± 1,038.19	1,520.65 ± 1,040.91	
Median [min-max]	824.50 [2.00,3632.00]	1940.00 [80.00,4037.00]	1,523.50 [2.00,4037.00]	
Vital status				**<0.001**
Alive	55 (42.97%)	90 (70.31%)	145 (56.64%)	
Dead	73 (57.03%)	38 (29.69%)	111 (43.36%)	
FG risk score				**<0.001**
Mean ± SD	1.91 ± 1.20	-0.57 ± 1.10	0.67 ± 1.69	
Median [min-max]	1.64 [0.59,7.31]	-0.21 [-5.77,0.59]	0.59 [-5.77,7.31]	

Notes: WBC, white blood cell; BM, bone marrow; PB, peripheral blast; ELN, European LeukemiaNet; FAB, French–American–British; NOS, not otherwise specified; CNS, central nervous system; MRD, measurable residual disease; HSCT, hematopoietic stem-cell transplantation; SD, standard deviation.

### Association of FGL risk score with the intermediate-risk subgroup of 2017 ELN is defined

**FIGURE 7:**
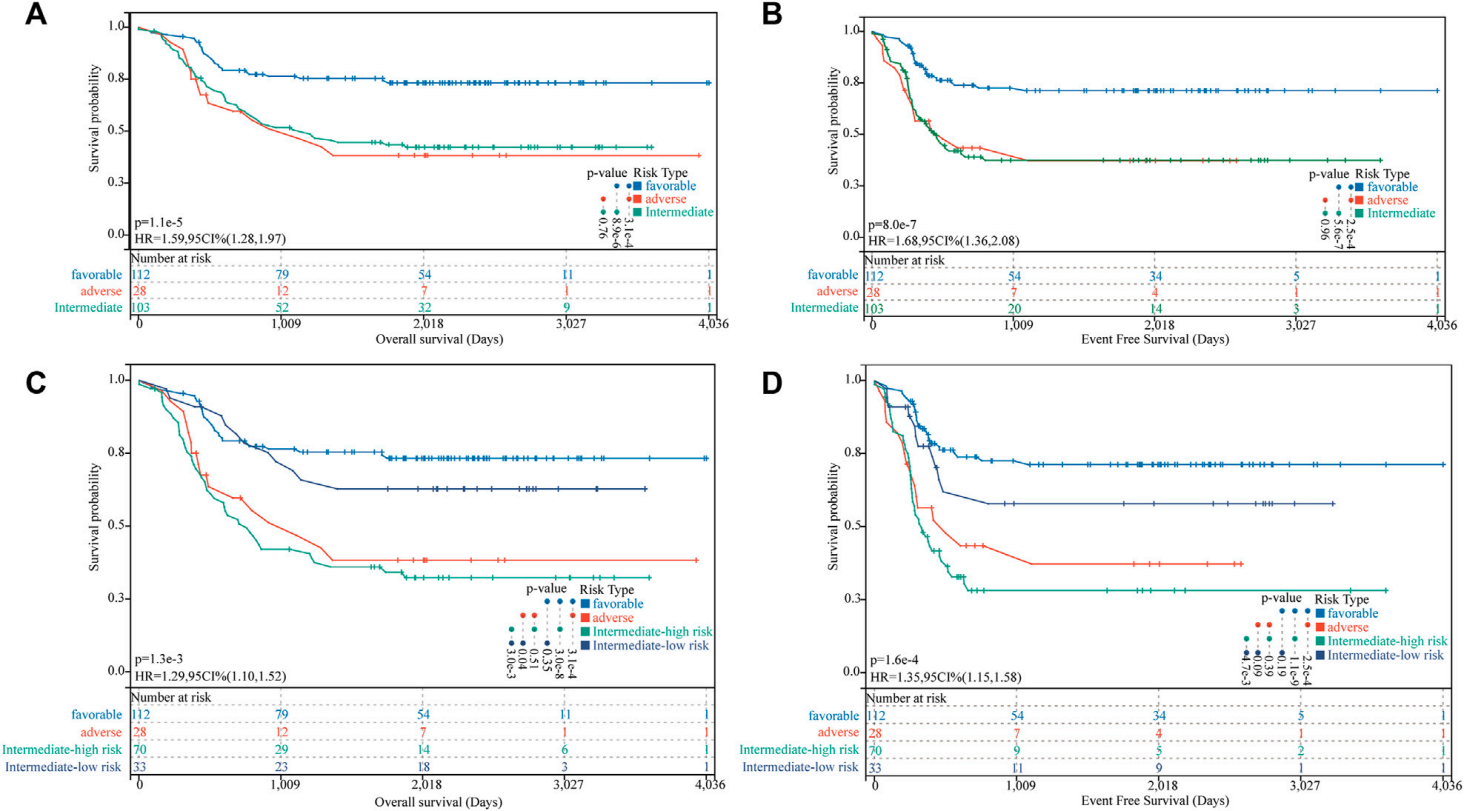
Incorporation of the FGL prognostic model in the 2017 ELN risk classification system. **(A,B)** Kaplan–Meier survival curves for OS and EFS of P-AML patients in the TARGET-combined cohort stratified according to the 2017 ELN classification system. **(C,D)** Patients in the 2017 ELN intermediate-risk subgroup could be well risk-stratified by the FGL risk score.

We identified 112 (43.75%), 103 (40.23%), and 28 (10.94%) patients in the TARGET-combined cohort classified as favorable-, intermediate-, and adverse-risk groups, respectively, according to the 2017 ELN classification system. Our results validated the prognostic significance of the revised 2017 ELN classification system in the TARGET-combined cohort (*p* < 0.001) (Figures 7A,B). Individuals in the favorable group defined by the 2017 ELN classification system had significantly better OS. However, no significant prognostic difference between the intermediate and adverse groups was found (*p* = 0.76 for OS; *p* = 0.96 for EFS). Then, in the 2017 ELN intermediate-risk subgroup of the TARGET-combined cohort (*N* = 103), we found 70 patients grouped with a high FGL risk score and 33 patients grouped with a low FGL risk score, which could be well risk-stratified by the FGL scoring system (*p* = 0.003 for OS; *p* = 0.0047 for EFS, Figures 7C,D).

### Functional analysis and immune characteristics of high-risk and low-risk groups

**FIGURE 8:**
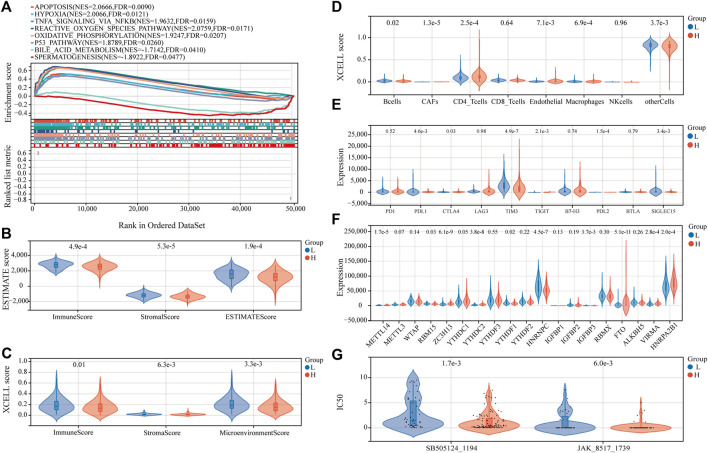
Estimation of functional and immune statuses in the high- and low-risk group using expression data in the TARGET-combined cohort. **(A)** Gene set enrichment analysis for ferroptosis-associated signatures. Violin plots of tumor purity for the low- and high-risk groups according to the **(B)** ESTIMATE and **(C)** xCell tool. **(D)** Violin plots of immune cell abundance in the high-risk and low-risk groups of P-AML patients using the EPIC tool. **(E)** Violin plots of the expression of immune checkpoints between the high- and low-risk groups. **(F)** Violin plots of the expression of m6A-associated genes in the high- and low-risk groups. **(G)** Violin plots of the predicted IC_50_ to SB505124_1194 and JAK_8517_1739 in the high- and low-risk groups.

Examining the molecular trends of divergence across two risk groups using GSEA revealed 8 hallmark gene sets significantly perturbed (Figure 8A, and detailed results for 50 hallmark gene sets are shown in Supplementary Table S6). Six tumor-related hallmarks, apoptosis, hypoxia, TNFA signaling *via* NFKB, reactive oxygen species pathway, oxidative phosphorylation, and *p53* pathway, were significantly enriched in the low-risk group, while bile acid (BA) metabolism pathway and spermatogenesis were found significantly enriched in the high-risk group.

To determine whether the FGL risk score was related to tumor immunity, we next evaluated the correlation between FGL risk and immune score and immune cell infiltration. We observed significantly higher immune score, microenvironment score, stromal score, and ESTIMATE score in the low-risk group (Figure 8B and Figure 8C; all *p* < 0.05). Strong relationship between infiltration levels of several immune cells and FGL risk score has also been established. As shown in Figure 8D, infiltration levels of cancer-associated fibroblasts (CAFs), CD4_T cells, and endothelial were significantly upregulated in the high-risk group, while infiltration levels of B cells, macrophages, and other cells were significantly upregulated in the low-risk group (all *p* < 0.05). Notably, we observed a statistically significant difference between the two groups in terms of the expression of several important immune checkpoint genes. The expression of *PDL1*, *CTLA4*, *TIGIT*, and *PDL2* were more highly expressed in the high-risk group (all *p* < 0.05). However, a substantial increase in the expression of *TIM3* was found in the low-risk group (*p* < 0.0001) (Figure 8E).

In addition, we investigated the expression of m6A-related genes between the two risk groups, and the results showed that most of them were more highly expressed in the high-risk group (all *p* < 0.05) (Figure 8F). Significant correlations were also identified for the expression levels of the 22 ferroptosis-related signatures and m6A-related genes (Supplementary Figure S4). According to m6A2Target, a comprehensive database for the target gene of writers, erasers, and readers (WERs) of m6A modification in a cancer cell line (Deng et al., 2021), 13 out of the 22 ferroptosis-related signatures in the FGL model were potential target genes of WERs of m6A modification in the leukemia cell line (Supplementary Table S7).

### Anticancer drug sensitivity analysis

Sensitivity to 198 anticancer drugs was compared between the high- and low-risk groups to provide potential treatment guidance for P-AML patients. The results for 153 drugs were not considered for differences analysis because more than 20% of the samples were missing from the predicted IC_50_ values. Since the predicted values of the samples differ significantly, differences analysis was applied after the removal of potential outliers (extremely high IC_50_ values). ROUT method was used for outlier identification (setting Q to 5%) (Motulsky and Brown, 2006). The results demonstrated that the IC_50_ values of SB505124_1194 and JAK_8517_1739 were significantly lower in patients within the FGL high-risk group, which implies that patients within the FGL high-risk group might benefit from treatment using SB505124_1194 and JAK_8517_1739 (Figure 8G).

## Discussion

Development of a reliable and applicable prognostic model for long-term survival prediction, risk stratification, and helping with therapeutic decision-making in AML is a far-reaching event, especially for pediatric AML patients.

In our study, we explored the role of ferroptosis-related signatures, which includes ferroptosis-related mRNAs and correlated lncRNAs in P-AML. A new model for prognosis prediction of P-AML was established with 22 signatures associated with ferroptosis in the discovery cohort of the TARGET AML program and further validated in the validation cohort and TARGET-combined cohort (all *p* < 0.05). Additionally, the FGL prognostic model was identified as the only independent prognostic factor for P-AML, irrespective of the well-known 2017 ELN classification system and other AML-related cytogenetic changes and gene mutations. Furthermore, substantial differences in the TME, functionally enriched pathways, expression profiles of immune checkpoint genes, and m6A-associated genes were identified between the low- and high-risk groups. To our knowledge, this is the first study to elucidate the prognostic impact of ferroptosis-related signatures on pediatric AML patients and suggest the great potential roles of ferroptosis in P-AML.

A question that cannot be ignored now is whether this FGL risk model provides additional prognosis value, or it contradicts the existing molecular risk factor or risk classification system. We found that low-risk factors (inv (16) mutation, FAB M4 subtype, and favorable subtype of the 2017 ELN classification system) (Thomas et al., 2009) were associated with low FGL risk, and similarly, high-risk factors (WT1 mutation, adverse subtype of the 2017 ELN classification system) (Rampal and Figueroa, 2016) were associated with high-risk scores. In our study, 2017 ELN classification system showed great prognostic significance in pediatric patients with AML and assigned 40.23% of individuals to the intermediate-risk group, consistent with the previously reported number of 50% (Grimwade et al., 2010; Wang et al., 2017), further supporting the need for subsequent stratification for this subgroup. The novelty introduced by our work is that the FGL prognostic model could well dichotomize the 2017 ELN intermediate-risk subgroup into two groups with distinct prognoses. At the same time, our model presented with the highest AUC value compared with other prognostic models established or validated in the TARGET cohort. Collectively, our FGL risk model had greater prognostic value and could be an important supplement to the application of the 2017 ELN classification system in P-AML.

There are also numerous existing models for adult AML early prediction or risk stratification, which have been compared to the FGL model corresponding to different aspects of modeling (Supplementary Table S8). For the immune risk score model proposed for adult AML (Wang Y. et al., 2021), we did not succeed in validating it in the TARGET dataset (data not shown). The inconsistency might be induced by the differences in the immune microenvironment of AML tumors between adults and children. A gene mutation-based model proposed additional genetic markers that might refine the current ELN classification (Eisfeld et al., 2020), which was quite promising since somatic mutations are more stable relative to RNA levels. We expect to see further internal and external validation of this model. Two outstanding models were also presented in 2018 for early prediction of AML, which had fundamental clinical prevention value for predicting AML in healthy people over 65 (Abelson et al., 2018). Considering the low prevalence of AML in children (Puumala et al., 2013), risk prediction might be more important than disease prediction for P-AML patients.

Recent results reveal that the immune system may function in part through ferroptosis to prevent tumorigenesis (Wang et al., 2019). According to the immune score analysis, the low-risk group was consistent with a longer OS rate and higher immune score, suggesting that high immune-related activity might result in a better prognosis in P-AML. This finding is consistent with a previous study on adult AML (Zeng et al., 2021). CD4_T cells can differentiate into a multitude of effector cells depending on the antigens present within the microenvironment (Zhu et al., 2010). CD4_T regulatory cells (Tregs) are a major subset of CD4_T cells, which have been reported to suppress anti-tumor immune effector responses in the TME and may be recruited and exploited by leukemic cells to evade immune surveillance (Ustun et al., 2011; Tay et al., 2021). Immune infiltration analysis indicated the high-risk group expressed significant enrichment of CD4_T cells. It provides further evidence that an immune suppressive environment presenting with a low immune score might correlate with poor prognosis in the high-risk group.

In the past few years, major efforts have been made to develop immune therapies for the treatment of AML patients. Several clinical trials with the aim to improve the survival of AML patients are ongoing, with immune-based therapeutic modalities such as monoclonal antibodies, T cell engagers, adoptive T-cell therapy, adoptive-NK therapy, checkpoint blockade *via* PD-1/PD-L1, CTLA4, and newer target such as TIM3 (Isidori et al., 2021). Considering the inhibition of the PD-1/PD-L1 axis demonstrated antileukemic activity and wide-spread expression of *PDL1* (Giannopoulos, 2019), patients in the high-risk group with higher levels of *PDL1, PDL2,* and *TIGHT* might be more suitable for treatment using anti-PD-L1 immune checkpoint inhibitor (ICI) approved in multiple solid tumors, Avelumab for example (Saxena et al., 2021). *TIM3* (T cell immunoglobulin and mucin domain-3) is an ideal target for selectively killing LSCs but not normal hematopoietic stem cells (HSCs) in most human AML cells, and it was significantly upregulated in the low-risk group, suggesting that patients in the low-risk group might benefit from anti-TIM3 antibodies (Wang Z. et al., 2021). In addition, providing potential immune therapy guidance, we also identified the correlation relationship between FGL risk score and anticancer drug sensitivity. However, the present finding was based on the predicted algorithm in cell lines, further validation with preclinical studies is warranted. In our study, the expression of identified FGL-related signatures was found significantly correlated with many m6A-regulator genes, and 13 out of them were identified as potential target genes of WERs of m6A modification in a leukemia cell line, this is in line with previous analysis showing that a wide-ranging connection was found between m6A methylation and ferroptosis using 31 cancer type-specific datasets in TCGA (Zhang, 2021). Moreover, the majority of m6A-regulator genes were found with higher expression in the high-risk group than those in the low-risk group, implying that a combination strategy of RNA epigenetics and ferroptosis therapies might benefit more with comprehensive consideration of the expression pattern of the specific m6A-regulator.

GSEA analysis indicated that the low-risk group might be protected from a high level of ferroptosis, or apoptosis-induced cancer cell death or through the function of *NOX1* and *ZEB1* in the *p53* and ROS pathway. Bile acids (BAs) are well known as chemical chaperones to reduce endoplasmic reticulum stress in hematopoietic stem cells (Oguro, 2019). Recently, BA was reported to play a key role in the reconstitution of hematopoiesis and BA levels in the blood of pediatric cancer patients and mice treated with chemotherapeutic agents were increased in synchrony with an early proliferation of bone marrow cells and recovery from myelosuppression (Sigurdsson et al., 2020). BA metabolism pathway was found significantly enriched in the high-risk group, proving further evidence that dysregulated cholesterol homeostasis might result in ferroptosis resistance and promote tumorigenicity and metastasis in cancer (Liu et al., 2021).

Furthermore, several limitations of the FGL prognostic model need to be noticed. This model is generated from bioinformatics analysis and has been validated by limited additional cohorts. Furthermore, external validations with a large patient population are certainly warranted in the near future. *SLC7A11* (solute carrier family 7 member 11) gene in the model was found as the direct protein target of ferroptosis agonists (erastin) according to experimental evidence from The Cancer Therapeutics Response Portal (CTRP) database (http://portals.broadinstitute.org/ctrp/) (Rees et al., 2016). However, molecular mechanisms and potential for therapeutic targets of the ferroptosis-related signatures on P-AML need further study.

In conclusion, we established a concise prognostic model composed of 22 ferroptosis-related signatures for predicting the prognosis of P-AML patients. The high-risk score was an independent poor prognostic parameter and influences the immune status, expression level of immune checkpoint genes, and enriched tumor-related pathways, thereby providing new evidence for immune therapy for P-AML. Furthermore, the FGL risk score further refines the predicted clinical outcomes of the 2017 ELN risk system by sub-dividing the intermediate-risk patients.

## Data Availability

The original contributions presented in the study are included in the article/Supplementary Material; further inquiries can be directed to the corresponding author.
