# Supplementary material for: Ferroptosis-related gene signature predicts the clinical outcome in pediatric acute myeloid leukemia patients and refines the 2017 ELN classification system
Source: Front Mol Biosci. 2022 Aug 11;9:954524. doi: 10.3389/fmolb.2022.954524 (PMC9403410; doi:10.3389/fmolb.2022.954524)
Supplement: Supplementary file 1 [file DataSheet1.pdf]

Supplementary Material

Supplementary Note 1:

Tumor immune microenvironment (TME) analysis

Estimate the Proportion of Immune and Cancer cells (EPIC) was applied for estimating the infiltration ratio of eight type of immune cells included B cells, CD4+ T cells, CD8+ T cells, Endothelial, Macrophages, NK cells, cancer-associated fibroblasts (CAFs) and other cells. We explored the difference in the expression of 10 potential immune checkpoint genes between groups included programmed cell death protein 1 (PD1), programmed cell death protein ligand 1 (PDL1), programmed cell death protein ligand 2 (PDL2), cytotoxic T-lymphocyte-associated protein 4 (CTLA4), Lymphocyte Activation Gene 3 Protein (LAG3), T-Cell Membrane Protein 3 (TIM3), V-Set And Immunoglobulin Domain-Containing Protein 9 (TIGHT), CD276 Antigen (B7-H3), B- And T-Lymphocyte-Associated Protein (BTLA) and sialic acid binding Ig like lectin 6 (SIGLEC6).

Supplementary Note 2:

Five-fold cross-validation

Given the limited sample size, a 5-fold cross-validation in TARGET-combined cohort was conducted to avoid overfitting and this analysis was based on R language (version 3.4.3; https://www.r-project.org). Briefly, we split data into five "folds" (equally sized groups). Then, withholding one-fold, we re-estimated predictive power of model on withheld group (AUC). Small average bias across 5-fold of cross-validation between discovery AUC values and validation AUC values indicate stable predictive accuracy of identified prognostic model.

Word cited: Hastie T, Tibshirani R, Friedman JH. The Elements of Statistical Learning: Data Mining, Inference, and Prediction. Springer, 2009.

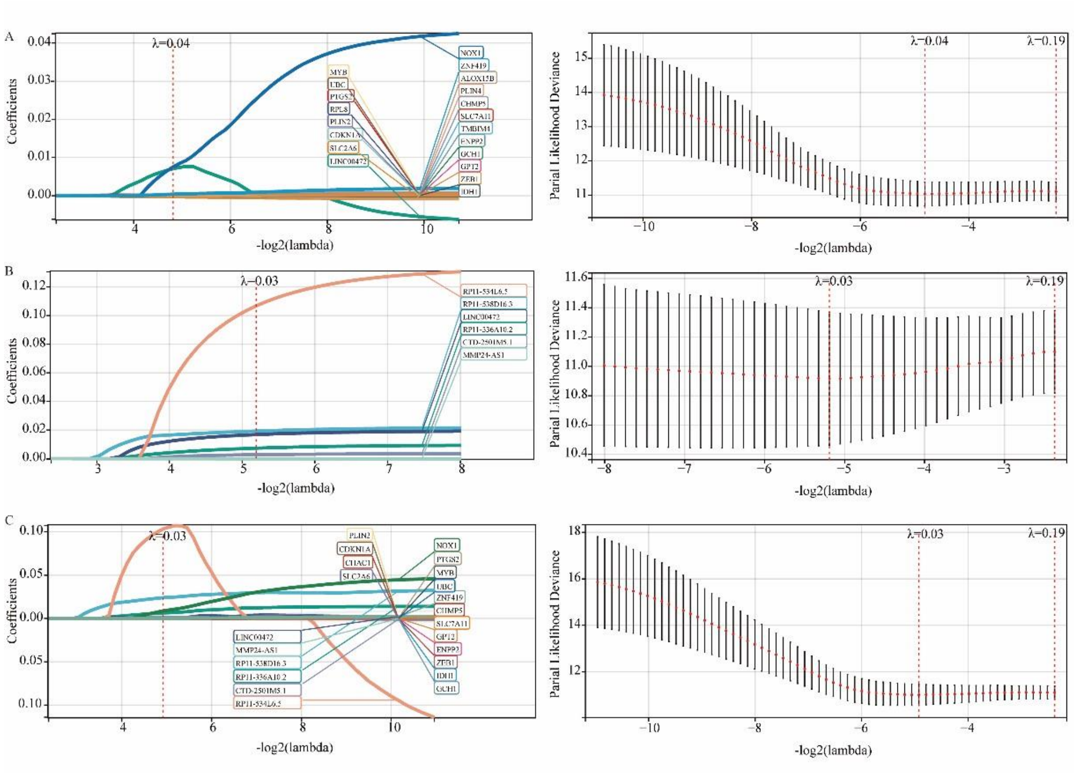

Figure S1. Prognostic models construction. Lasso regression analysis was used to construct prognostic models using prognostic (A) ferroptosis-related genes (FG), (B) ferroptosis-related lncRNAs (FL) and (C) FGL (FG and FL combined) from univariate Cox regression analysis, respectively. Coefficients of selected features are shown by lambda parameter (left)；Partial likelihood deviance versus log (λ)was drawn using LASSO Cox regression model (right).

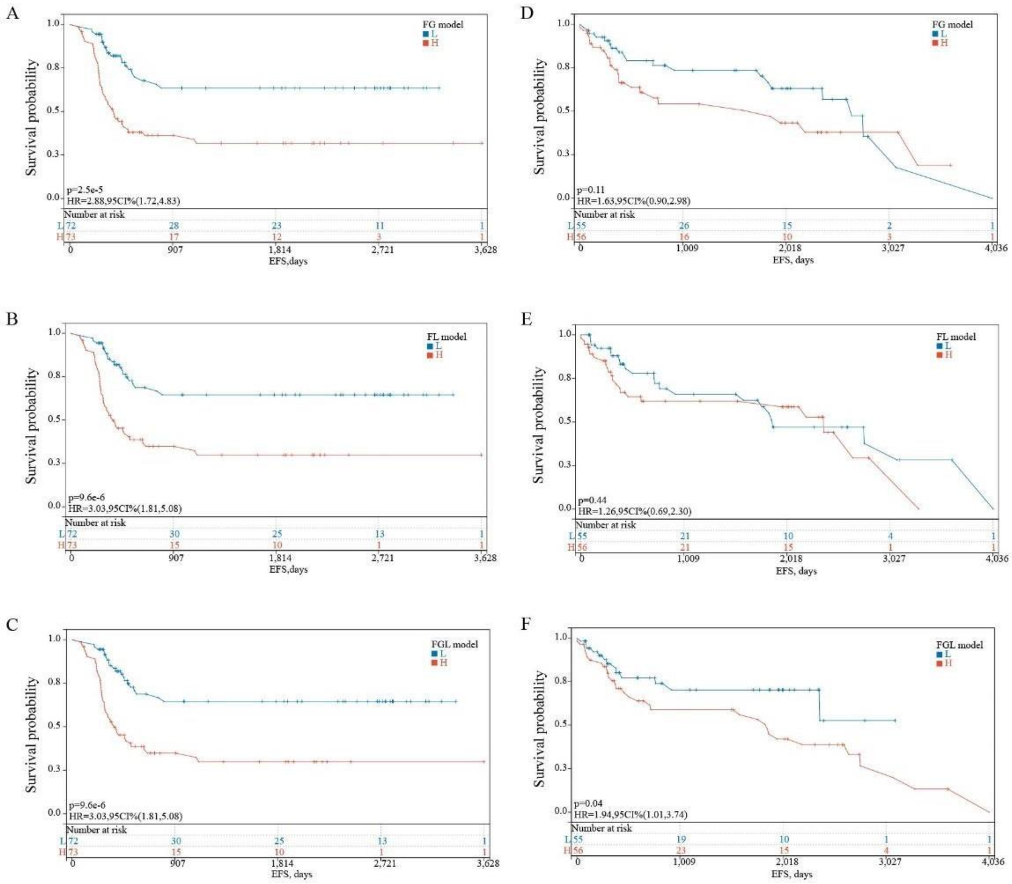

Figure S2.The predictive ability of the FG、FL and FGL prognostic model for the EFS of P-AML. Based on the median risk score of (A、D) FG, (B、E) FL and (C、F) FGL model, patients were divided into high-risk group and low-risk group. (left) Kaplan–Meier curve of the high-risk and low-risk groups of TARGET-discovery cohort. (right) Kaplan–Meier curve of the high-risk and low-risk groups of TARGET-validation cohort.

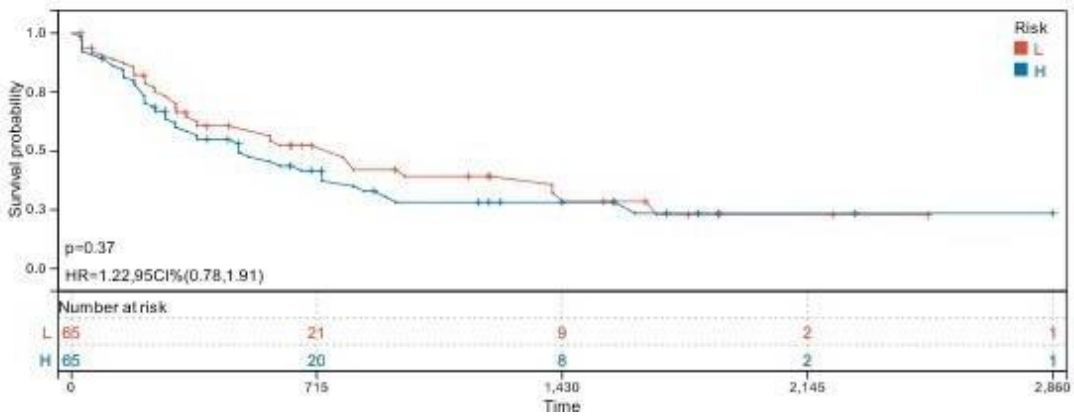

Figure S3. Validation of FGL risk model in adult AML patients (TCGA-LAML cohort)

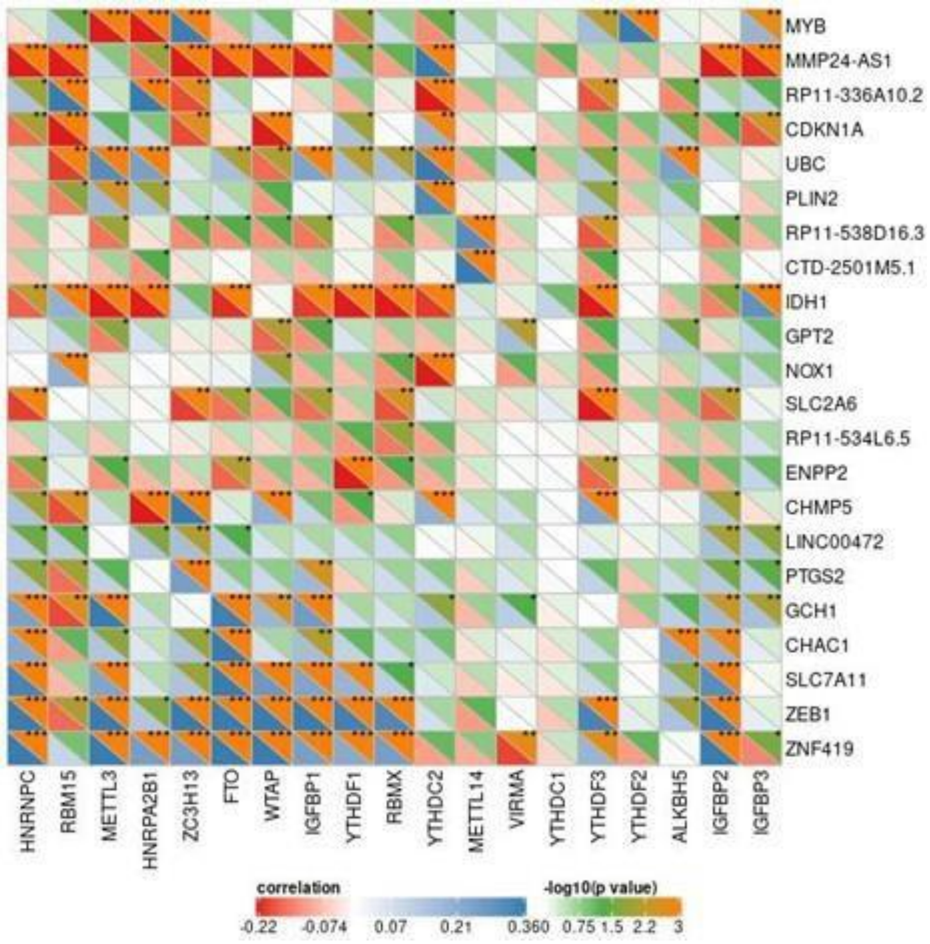

Figure S4. Heat map showing the correlations between FGL risk score and m6A regulators.Notes：*：P<0.05; **：P<0.01; ***：P<0.001.

Table S1. 259 ferroptosis-related genes from FerrDb database.

Gene Symbol

RPL8	TNFAIP3	OXSR1	MAPK14	ZFP36	NGB	KEAP1
IREB2	TLR4	SELENOS	LINC00472	PROM2	YWHAE	HMOX1
ATP5MC3	ATF3	ANGPTL7	PRKAA2	CHMP5	GABPB1	ATG5
CS	ATM	DDIT4	PRKAA1	CHMP6	AURKA	ATG7
EMC2	YY1AP1	LOC284561	ELAVL1	CAV1	MIR4715	NCOA4
ACSF2	EGLN2	ASNS	BAP1	GCH1	RIPK1	ALOX12
NOX1	MIOX	TSC22D3	ABCC1	PTGS2	PRDX1	ALOX12B
CYBB	TAZ	DDIT3	MIR6852	DUSP1	MIR30B	ALOX15

NOX3	MTDH	JDP2	ACVR1B	NOS2	SLC2A6	ALOX15B
NOX4	IDH1	SLC1A4	TGFBR1	NCF2	SLC2A8	ALOXE3
NOX5	SIRT1	PCK2	EPAS1	MT3	SLC2A12	PHKG2
DUOX1	FBXW7	TXNIP	HILPDA	UBC	GLUT13	FANCD2
DUOX2	PANX1	VLDLR	HIF1A	ALB	SLC2A14	FTMT
G6PD	DNAJB6	GPT2	IFNG	TXNRD1	EIF2AK4	HSPA5
PGD	BACH1	PSAT1	ANO6	SRXN1	TFAP2C	ATF4
VDAC2	LONP1	LURAP1L	LPIN1	GPX2	SP1	HELLS
PIK3CA	SLC7A11	SLC7A5	HMGB1	BNIP3	HBA1	SCD
FLT3	GPX4	HERPUD1	CDKN2A	JUN	NNMT	FADS2
SCP2	AKR1C1	XBP1	PEBP1	CA9	PLIN4	SRC
TP53	AKR1C2	ZNF419	SOCS1	TMBIM4	HIC1	STAT3
ACSL4	AKR1C3	KLHL24	CDO1	PLIN2	STMN1	PML
LPCAT3	RB1	TRIB3	MYB	MIR212	RRM2	MTOR
NRAS	HSPB1	ZFP69B	MAPK8	Fer1HCH	CAPG	KIM-1
KRAS	HSF1	ATP6V1G2	MAPK9	AIFM2	HNF4A	IL6
HRAS	GCLC	VEGFA	CHAC1	LAMP2	STEAP3	CXCL2
TF	NFE2L2	GDF15	MAP1LC3A	CISD2	DRD5	RELA
TFRC	SQSTM1	TUBE1	GABARAPL2	MIR9-1	DRD4	HSD17B11
TFR2	NQO1	ARRDC3	GABARAPL1	MIR9-2	MAP3K5	AGPAT3
SLC38A1	FTH1	CEBPG	ATG16L1	MIR9-3	SLC2A1	SETD1B
SLC1A5	MUC1	SNORA16A	WIPI1	CBS	BECN1	FTL
GLS2	SLC3A2	RGS4	WIPI2	ISCU	FH	MAFG
GOT1	MT1G	BLOC1S5-TXNDC5	SNX4	ACSL3	SLC2A3	IL33
CARS1	SLC40A1	LOC390705	ATG13	OTUB1	ATG4D	HAMP
ALOX5	CISD1	EIF2S1	ULK2	CD44	ENPP2	SAT1
EGFR	MAPK3	BID	DPP4	G6PDX	LINC00336	CDKN1A
MAPK1	ATG3	ZEB1	ACO1	ULK1	BRD4	MIR137
PRDX6	MIR17	SESN2	NF2	ARNTL	NFS1	TP63

Table S2. 39 ferroptosis-related genes and 8 ferroptosis-related lncRNAs with significant prognostic value identified with univariate COX analysis

	symbol	variable	coef	HR	HR.95L	HR.95H	pvalue
39 ferroptosis-related genes identified with univariate Cox regression analysis							
1	TMBIM4	ENSG00000155957.15	0.0001868	1.000186796	1	1.000373	0.0493596
2	LINC00472	ENSG00000233237.5	0.0226223	1.0228801	1.01013	1.035791	0.0004079
3	RPL8	ENSG00000161016.14	-1.00E-05	0.999989951	0.99998	0.999996	0.0010664
4	PLIN4	ENSG00000167676.3	0.0010895	1.001090139	1.00013	1.002055	0.0266454
5	SLC2A3	ENSG00000059804.14	-1.24E-05	0.999987649	0.99998	0.999999	0.0406177
6	VDAC2	ENSG00000165637.12	-8.23E-05	0.999917683	0.99985	0.999988	0.0215495
7	PRKAA1	ENSG00000132356.10	0.0001064	1.000106399	1	1.000213	0.0494443
8	CHMP5	ENSG00000086065.12	0.0003886	1.000388671	1.00013	1.000646	0.0030866
9	TUBE1	ENSG00000074935.12	0.0010667	1.001067273	1.00041	1.00172	0.0013503
10	ZFP36	ENSG00000128016.5	-2.82E-06	0.999997182	0.99999	1	0.0239063
11	MTOR	ENSG00000198793.11	0.0002021	1.000202165	1.00001	1.000395	0.0402505
12	CDKN1A	ENSG00000124762.12	-4.42E-05	0.999955817	0.99992	0.999989	0.0092811
13	PTGS2	ENSG00000073756.10	2.68E-05	1.000026777	1	1.00005	0.0215303
14	CHAC1	ENSG00000128965.10	0.0018702	1.001871995	1.00083	1.00292	0.0004521
15	UBC	ENSG00000150991.13	-1.68E-05	0.999983227	0.99997	0.999994	0.0017614
16	ALOX15B	ENSG00000179593.14	0.0015045	1.001505626	1.00013	1.002879	0.031413
17	HSF1	ENSG00000185122.9	-9.66E-05	0.999903372	0.99982	0.999984	0.0183031
18	RELA	ENSG00000173039.17	-5.29E-05	0.99994715	0.9999	0.999995	0.0317301
19	SLC7A11	ENSG00000151012.12	0.0004032	1.00040333	1.00017	1.000632	0.0005454
20	ACO1	ENSG00000122729.17	0.0007068	1.000707045	1.00004	1.001378	0.0388925
21	NOX1	ENSG00000007952.16	0.0312452	1.031738484	1.00092	1.063503	0.0434291
22	SAT1	ENSG00000130066.15	-7.26E-06	0.999992737	0.99999	0.999999	0.0321297
23	CD44	ENSG00000026508.15	-9.02E-06	0.999990978	0.99998	0.999997	0.0059017
24	SLC2A6	ENSG00000160326.12	-0.000696	0.999304616	0.99874	0.999871	0.0160641
25	GPX4	ENSG00000167468.15	-2.35E-05	0.999976467	0.99996	0.999996	0.0197725
26	GCH1	ENSG00000131979.17	0.0002433	1.000243318	1.00005	1.00044	0.0152728
27	PRDX6	ENSG00000117592.8	-4.59E-05	0.999954138	0.99992	0.999992	0.0184306
28	PLIN2	ENSG00000147872.8	-2.51E-05	0.999974907	0.99996	0.999994	0.0103785
29	ENPP2	ENSG00000136960.11	0.0002608	1.000260884	1.00006	1.000459	0.0098704
30	BID	ENSG00000015475.17	-0.000111	0.999889091	0.99978	0.999997	0.044864
31	MYB	ENSG00000118513.17	2.42E-05	1.000024219	1.00001	1.000043	0.0098654
32	GPT2	ENSG00000166123.12	0.0002283	1.000228316	1.00007	1.000384	0.0040983
33	ATF4	ENSG00000128272.13	-1.45E-05	0.999985522	0.99997	0.999997	0.0117811
34	IDH1	ENSG00000138413.12	0.0002385	1.000238572	1.00009	1.000385	0.0014059

35	FTL	ENSG00000087086.12	-1.88E-06	0.999998121	1	0.999999	0.0037155
36	FTH1	ENSG00000167996.14	-3.66E-06	0.999996339	0.99999	0.999999	0.0058228
37	ZEB1	ENSG00000148516.20	0.0001336	1.00013357	1.00002	1.000248	0.0225626
38	GOT1	ENSG00000120053.10	0.0007171	1.000717363	1.00009	1.00135	0.0261435
39	ZNF419	ENSG00000105136.18	0.0010586	1.001059209	1.00006	1.002057	0.0373472

8 prognostic ferroptosis-related lncRNAs

	symbol	variable	coef	HR	HR.95L	HR.95H	pvalue
1	RP11-538D16.3	ENSG00000225982.1	0.0207205	1.020936645	1.0088	1.033221	0.0006854
2	RP11-336A10.2	ENSG00000226647.2	0.0113152	1.011379468	1.00074	1.022132	0.0359803
3	LINC00472	ENSG00000233237.5	0.0226223	1.0228801	1.01013	1.035791	0.0004079
4	RP11-45A17.4	ENSG00000273521.1	0.0065329	1.006554314	1.00223	1.010899	0.0029524
5	RP11-534L6.5	ENSG00000251413.1	0.2014742	1.22320469	1.07463	1.392327	0.0022942
6	CTD-2501M5.1	ENSG00000253507.4	0.0083568	1.008391852	1.00404	1.012759	0.0001505
7	MMP24-AS1	ENSG00000126005.14	-0.000157	0.999842642	0.99974	0.999942	0.0018505
8	RP11-257P3.3	ENSG00000253374.4	-0.007469	0.992559178	0.98539	0.999779	0.0434099

Table S3. 20 FG components, 6 FL components and 22 FGL components were screened out with LASSO Cox regression

20 FG components		6 FL components		22 FGL components	
Lambda=0.0356		Lambda=0.0274		Lambda=0.0330	
symbol	coefficient	symbol	coefficient	symbol	coefficient
TMBIM4	0.000159146	RP11-538D16.3	0.019229	RP11-538D16.3	0.024022
LINC00472	0.007088235	RP11-336A10.2	0.007291	RP11-336A10.2	0.006289
RPL8	-1.59E-06	LINC00472	0.016798	LINC00472	0.000452
PLIN4	4.76E-06	RP11-534L6.5	0.106714	RP11-534L6.5	0.103517
CHMP5	9.61E-05	CTD-2501M5.1	0.002749	CTD-2501M5.1	0.001274
CDKN1A	-6.29E-06	MMP24-AS1	-0.00013	MMP24-AS1	-0.000031
PTGS2	6.22E-06			CHMP5	0.000160
UBC	-4.78E-06			CDKN1A	-0.000009
ALOX15B	0.000399446			PTGS2	0.000014
SLC7A11	0.000143754			CHAC1	0.000286
NOX1	0.007555389			UBC	-0.000004
SLC2A6	-0.00028645			SLC7A11	0.000168
GCH1	0.000124285			NOX1	0.008705
PLIN2	-4.62E-07			SLC2A6	-0.000109

ENPP2	0.000179514	GCH1	0.000024
MYB	9.60E-06	PLIN2	-0.000002
GPT2	6.56E-05	ENPP2	0.000215
IDH1	6.86E-05	MYB	0.000010
ZEB1	4.42E-05	GPT2	0.000089
ZNF419	0.000424126	IDH1	0.000121
		ZEB1	0.000056
		ZNF419	0.000219

Table S4. The FG、FL and FGL risk score for each patient in TARGET-discovery cohort.

Tag	Time	Status	FG risk score	FL risk score	FGL risk score
TARGET-20-PADYIR-01R	585	1	1.170212	-0.23907	1.1325428
TARGET-20-PADZCG-02R	536	1	1.080784	-0.167701	1.0754599
TARGET-20-PAEAKL-03R	163	1	1.243492	-0.178986	1.3797754
TARGET-20-PAECCE-01R	147	1	0.8177509	-0.236429	0.6114915
TARGET-20-PAEERJ-01R	373	1	1.9586042	-0.176487	1.9250727
TARGET-20-PAEFGR-02R	4022	0	0.5222429	-0.223207	0.5962571
TARGET-20-PAEFGT-01R	315	1	1.5246632	-0.307654	1.7881934
TARGET-20-PAEIKD-01R	3600	0	0.7983753	-0.159828	0.4376546
TARGET-20-PAKERZ-01R	397	1	2.2319809	-0.173065	2.5556348
TARGET-20-PAKIWK-01R	112	0	1.1598832	0.3673576	1.0964953
TARGET-20-PAKIYW-01R	2965	0	1.2519295	-0.383393	0.9152751
TARGET-20-PAKLPD-02R	2707	0	-0.005102	-1.210291	-0.235904
TARGET-20-PAKSMZ-02R	1293	1	1.2749697	-0.129182	1.0924906
TARGET-20-PAKTCX-02R	1896	1	0.9109381	-0.270938	0.7666043
TARGET-20-PAKVGI-01R	1229	1	1.0588571	-0.009219	0.7915158
TARGET-20-PALGKX-03R	485	1	0.8620838	-0.37744	1.3550079
TARGET-20-PAMVKZ-03R	693	1	0.8402998	-0.368711	0.6896804
TARGET-20-PAMYAS-03R	3263	0	0.1289175	-1.025108	0.0800551
TARGET-20-PAMYGX-03R	3380	0	0.8517097	0.0741654	0.7458305
TARGET-20-PANAEV-03R	2767	0	0.0809392	-0.592303	0.1961959
TARGET-20-PANBZH-03R	462	1	0.2350385	-0.354031	0.4554655
TARGET-20-PANDER-02R	3291	0	-0.014619	-0.88374	-0.419895
TARGET-20-PANDIX-03R	3069	0	1.1113542	-0.297175	1.1157213
TARGET-20-PANFMG-04R	3253	0	0.4606208	-0.332905	0.5691753
TARGET-20-PANGCM-03R	3055	0	-0.532562	-1.553429	-0.704049
TARGET-20-PANGDN-05R	3063	0	1.1955203	-0.022192	1.1360441
TARGET-20-PANGJY-03R	2983	0	0.5364905	-1.345704	0.2420544
TARGET-20-PANGTF-04R	616	1	0.4754015	-0.33065	0.471925
TARGET-20-PANHYK-01R	1675	0	0.2672615	-0.323596	0.3494487

TARGET-20-PANINI-03R	3144	0	0.2321345	-1.508005	0.0221476
TARGET-20-PANKFZ-04R	3116	0	-0.803748	-1.898769	-1.065507
TARGET-20-PANKKE-03R	3149	0	0.0827249	-0.693259	-0.279529
TARGET-20-PANKNB-03R	443	0	0.2550348	-1.035332	0.0387861
TARGET-20-PANLIR-02R	3238	0	0.7015155	-0.147264	0.7250111
TARGET-20-PANLIZ-03R	784	1	-0.101443	-0.469336	-0.090063
TARGET-20-PANLJN-03R	2133	0	1.0982324	-0.490478	0.8684708
TARGET-20-PANLKB-03R	1985	0	-0.161329	-0.920358	-0.38283
TARGET-20-PANLLX-01R	3234	0	0.9784499	-0.126128	0.8616083
TARGET-20-PANLXM-03R	390	1	1.3868573	0.0713201	1.4341258
TARGET-20-PANNHB-03R	436	1	0.262034	-0.383865	0.1984607
TARGET-20-PANPKN-03R	3630	0	1.2143408	-0.25614	1.1560029
TARGET-20-PANPLS-04R	702	1	0.3729119	-0.047261	0.5198535
TARGET-20-PANPTM-02R	3130	0	0.6366736	-0.653444	0.5513579
TARGET-20-PANSBH-05R	813	1	0.7924267	0.1408859	1.0273581
TARGET-20-PANSJB-03R	422	1	2.1824442	-0.415617	2.2719929
TARGET-20-PANTNA-02R	1152	1	0.4387485	-0.895452	0.2410052
TARGET-20-PANTPW-02R	2946	0	0.736535	-0.916096	0.5840952
TARGET-20-PANUTB-05R	925	1	0.4071432	-0.247109	0.3849559
TARGET-20-PANUUA-02R	1767	1	0.1385958	-0.619432	0.0502771
TARGET-20-PANVGE-02R	2819	0	-0.989743	-2.250676	-0.94805
TARGET-20-PANVGP-04R	2688	0	-0.12721	0.0750277	0.1318253
TARGET-20-PANWHP-02R	2738	0	-0.139998	-0.727901	-0.011392
TARGET-20-PANYNR-03R	2952	0	0.6034407	-0.345352	0.5641046
TARGET-20-PANYSN-04R	914	0	1.3717979	0.0528356	1.4681736
TARGET-20-PANZKA-02R	2824	0	0.4718747	-1.159069	0.2150364
TARGET-20-PAPAWN-02R	2605	0	0.3461274	-0.339465	0.4686632
TARGET-20-PAPBEJ-03R	404	1	1.2825137	0.2562002	1.2295492
TARGET-20-PAPVCN-03R	2592	0	-0.705902	-2.098625	-0.978211
TARGET-20-PAPVDV-01R	2688	0	0.543062	-0.16375	0.5609275
TARGET-20-PAPVZK-03R	2492	0	-0.405661	-1.078775	-0.635999
TARGET-20-PAPWHS-03R	2687	0	-1.20675	-1.321068	-1.446025
TARGET-20-PAPWIU-03R	2829	0	-0.143536	-1.8878	-0.339946
TARGET-20-PAPWYK-03R	199	1	-0.126237	-0.950157	-0.179529
TARGET-20-PAPXRJ-01R	1248	1	1.3554603	-0.125732	1.43657
TARGET-20-PAPXWI-03R	880	0	-0.803998	-1.497308	-0.985923
TARGET-20-PARAJX-02R	2803	0	-0.143363	-1.128472	-0.269441
TARGET-20-PARANT-02R	2509	0	0.0792143	-0.461593	0.046432
TARGET-20-PARASV-03R	749	1	0.9418971	-0.427339	0.5599317
TARGET-20-PARBFJ-02R	2387	0	0.9275454	-0.548342	0.5275718
TARGET-20-PARBIU-03R	917	1	0.5962815	-0.0831	0.4393297
TARGET-20-PARBRA-03R	1362	1	0.8291286	0.7496224	1.9208403
TARGET-20-PARCCH-03R	228	1	4.0493414	2.4093281	4.2414657
TARGET-20-PARCUK-03R	279	1	2.1230709	1.7846696	3.2030987

TARGET-20-PARCZL-02R	2695	0	0.1475422	-0.002182	0.1851137
TARGET-20-PARDDY-05R	766	1	1.1354975	-0.195882	1.1862232
TARGET-20-PARDMG-03R	413	1	0.6749663	-0.282519	0.610434
TARGET-20-PARENB-03R	314	0	0.7091517	-0.257578	0.755274
TARGET-20-PARFAL-05R	378	1	1.7019392	-0.104279	1.3986395
TARGET-20-PARGVC-03R	295	1	-0.536467	-1.571092	-0.788769
TARGET-20-PARHVK-04R	2516	0	1.1393513	-0.377372	1.3036361
TARGET-20-PARIHK-03R	923	1	1.542991	-0.024908	1.7211952
TARGET-20-PARIMT-01R	2476	0	0.5624372	-0.455123	0.5406341
TARGET-20-PARIZR-02R	2457	0	0.9716079	-0.320437	1.1971046
TARGET-20-PARJCR-01R	468	1	1.5806433	-0.592072	1.2846276
TARGET-20-PARJYP-01R	1798	0	0.344711	-1.017325	0.187907
TARGET-20-PARKCX-03R	2760	0	0.9833056	-0.224194	0.7380707
TARGET-20-PARMZF-02R	2193	0	0.0945835	-0.532054	-0.095304
TARGET-20-PARPDS-01R	836	1	0.7369587	-0.204863	0.7056733
TARGET-20-PARPWL-03R	1199	0	0.4164409	-0.796098	0.3759236
TARGET-20-PARSAN-01R	870	1	1.2709499	-0.105431	1.1123567
TARGET-20-PARSHM-02R	2450	0	0.6480329	-0.26944	0.5365496
TARGET-20-PARTAL-02R	531	1	0.8958545	-0.33339	0.4576577
TARGET-20-PARUDL-01R	2411	0	-0.223521	-0.33244	0.0435367
TARGET-20-PARUNX-04R	2364	0	1.0483588	-0.169444	0.8945538
TARGET-20-PARUTH-04R	1412	1	0.0322016	-0.53394	-0.187355
TARGET-20-PARUUB-02R	1882	0	0.9499636	-0.22801	1.4015172
TARGET-20-PARUWX-02R	439	1	1.0677487	0.0161869	1.5272914
TARGET-20-PARVAI-02R	429	1	1.717279	0.210899	1.8197633
TARGET-20-PARWDZ-01R	321	1	1.3044167	0.1542425	1.5229075
TARGET-20-PARXBT-02R	2246	0	0.7805214	0.1190437	0.9983689
TARGET-20-PARXMP-02R	1903	0	0.8622023	0.079888	0.7465315
TARGET-20-PARXNG-01R	2246	0	0.8947335	-0.125261	0.9696417
TARGET-20-PARYFN-01R	2203	0	0.4088676	-0.143143	0.5340792
TARGET-20-PARYGA-03R	2406	0	0.7856079	-0.363746	0.6032598
TARGET-20-PARYVW-01R	2317	0	0.630039	0.1629089	0.9565614
TARGET-20-PARZUU-04R	2168	0	0.8639189	-0.163581	0.8611112
TARGET-20-PARZWH-03R	2151	0	0.8721151	-0.745505	0.831644
TARGET-20-PASBHI-01R	2342	0	-0.698572	-1.265641	-0.563067
TARGET-20-PASBPK-02R	2117	0	0.5737894	-0.666639	0.294859
TARGET-20-PASCFW-01R	2205	0	1.0937762	-0.282461	1.1520212
TARGET-20-PASCGR-02R	702	1	1.3485033	-0.116809	1.3889538
TARGET-20-PASFEW-05R	313	1	1.9224138	0.6118124	1.6691845
TARGET-20-PASGGK-01R	2025	0	0.8877098	0.0181423	0.94481
TARGET-20-PASGMZ-01R	789	1	1.741734	0.0705223	1.6773935
TARGET-20-PASGWH-04R	1214	1	1.4313556	-0.399543	1.3974488
TARGET-20-PASHBI-01R	1335	0	0.8753316	0.2292776	1.0685165

TARGET-20-PASHYZ-03R	433	1	0.3393422	-0.460572	0.4662091
TARGET-20-PASIBG-01R	1094	1	0.7092621	0.0833099	0.7791627
TARGET-20-PASJTM-01R	1953	0	1.3336858	0.9737416	1.6162759
TARGET-20-PASLSD-01R	2126	0	0.8099901	-0.163622	0.7304015
TARGET-20-PASMGW-01R	286	1	1.2579204	0.4107994	1.6781576
TARGET-20-PASMHY-03R	592	1	0.4422483	-0.199348	0.4706554
TARGET-20-PASPGA-02R	1729	1	1.4100079	-0.239879	1.3932402
TARGET-20-PASPKE-04R	2026	0	0.0447143	-0.599955	0.0649358
TARGET-20-PASPLU-02R	351	1	2.8265894	-0.097981	2.9581237
TARGET-20-PASPTM-03R	1900	0	0.6161748	-0.043916	0.5691392
TARGET-20-PASSSI-02R	712	1	0.916836	0.0590519	1.1440398
TARGET-20-PASTTW-01R	600	1	1.3391555	1.1378632	1.8039826
TARGET-20-PASTUH-01R	166	1	0.9622478	0.1129742	1.1224969
TARGET-20-PASVVS-03R	1968	0	1.2958029	-0.407211	1.1263026
TARGET-20-PASVYA-01R	630	1	1.9871436	0.1656581	1.7941591
TARGET-20-PASVYL-01R	286	1	2.3615148	1.6242763	3.2314025
TARGET-20-PASWAJ-04R	1712	0	1.5034332	-0.381774	1.3799706
TARGET-20-PASWAT-01R	342	1	1.4417622	-0.102657	1.7743512
TARGET-20-PASWLN-01R	614	1	1.4435632	-0.085091	1.4575564
TARGET-20-PASWPD-02R	689	1	1.7806262	-0.08878	1.8403167
TARGET-20-PASWPT-01R	518	1	1.0962915	-0.055049	1.170715
TARGET-20-PASXYG-05R	570	1	0.863454	-0.225947	1.1332127
TARGET-20-PASYJI-03R	1780	0	0.16663	-0.605195	0.1829995
TARGET-20-PASZLJ-01R	289	1	2.4234366	-0.005736	2.4828306
TARGET-20-PATDHA-01R	403	1	0.1992646	-1.185193	0.2483099
TARGET-20-PATDNN-03R	194	1	1.0082561	3.3842096	4.4948552
TARGET-20-PATELT-05R	1652	0	1.333799	-0.226118	1.258368
TARGET-20-PATIAK-01R	1464	0	0.0957134	-0.029707	0.017698
TARGET-20-PATJHJ-01R	607	1	1.8811722	0.299969	1.9215479

Table S5. Five-fold cross-validation to build predicting model

	AUC		
TARGET-discovery cohort	train	test	bias
	0.683477	0.795998	-0.112520792
	0.895003	0.751092	0.143910879
	0.75467	0.788414	-0.033743759

	0.655339	0.815556	- 0.160216799
	0.871375	0.745746	0.125629273
average	0.771973	0.779361	-0.00738824
		AUC	
TARGET-combined cohort	train	test	bias
	0.556834	0.741369	-0.18453527
	0.69493	0.715519	- 0.020589231
	0.783748	0.693108	0.090640626
	0.759436	0.696951	0.062485587
	0.774256	0.697237	0.07701845
	0.713841	0.708837	0.005004032

Bias equals training AUC value minus testing AUC. AUC, area under the receiver operatingcharacteristic curve

Table S6. GSEA analysis for 50 hallmark gene sets.

Term	ES	NES	p-value	FDR
**APOPTOSIS**	**0.5244**	**2.0666**	**0**	**0.009**
**HYPOXIA**	**0.5169**	**2.0066**	**0.002**	**0.0121**
**TNFA_SIGNALING_VIA_NFKB**	**0.6699**	**1.9632**	**0.0061**	**0.0159**
**REACTIVE_OXYGEN_SPECIES_PATHWAY**	**0.691**	**2.0759**	**0**	**0.0171**
**OXIDATIVE_PHOSPHORYLATION**	**0.6634**	**1.9247**	**0.0178**	**0.0207**
**P53_PATHWAY**	**0.4763**	**1.8789**	**0.002**	**0.026**
**BILE_ACID_METABOLISM**	**-0.4023**	**-1.7142**	**0**	**0.041**
**SPERMATOGENESIS**	**-0.4615**	**-1.8922**	**0**	**0.0477**
UV_RESPONSE_UP	0.4572	1.7304	0.01	0.0507
MITOTIC_SPINDLE	-0.4602	-1.7246	0.027	0.0514
ADIPOGENESIS	0.4678	1.7422	0.0293	0.0533
UV_RESPONSE_DN	-0.4116	-1.6262	0.0228	0.0561
IL2_STAT5_SIGNALING	0.445	1.7526	0.0116	0.0561
PI3K_AKT_MTOR_SIGNALING	0.4355	1.7019	0.0097	0.0564

ALLOGRAFT_REJECTION	0.4816	1.767	0.0199	0.0591
CHOLESTEROL_HOMEOSTASIS	0.4673	1.6669	0.033	0.0656
G2M_CHECKPOINT	-0.5257	-1.7379	0.0414	0.0711
E2F_TARGETS	-0.4936	-1.5146	0.1266	0.0856
MYC_TARGETS_V1	0.4957	1.5065	0.1361	0.1279
IL6_JAK_STAT3_SIGNALING	0.4639	1.5124	0.082	0.1319
DNA_REPAIR	0.4183	1.477	0.1155	0.1393
MTORC1_SIGNALING	0.3978	1.4418	0.1071	0.1395
APICAL_JUNCTION	0.3532	1.5131	0.0263	0.1402
MYOGENESIS	0.3517	1.4608	0.0499	0.1422
ESTROGEN_RESPONSE_LATE	0.3588	1.4465	0.0598	0.1433
INFLAMMATORY_RESPONSE	0.4272	1.5187	0.086	0.1465
FATTY_ACID_METABOLISM	0.3682	1.421	0.1205	0.1476
PEROXISOME	-0.3441	-1.3687	0.1104	0.1478
KRAS_SIGNALING_DN	-0.31	-1.3116	0.0713	0.1664
XENOBIOTIC_METABOLISM	0.3266	1.3417	0.0973	0.1963
COMPLEMENT	0.3547	1.3145	0.1588	0.2002
COAGULATION	0.3394	1.3239	0.1164	0.2013
ESTROGEN_RESPONSE_EARLY	0.3038	1.3	0.0916	0.2048
PROTEIN_SECRETION	-0.3515	-1.1974	0.283	0.2365
GLYCOLYSIS	0.3081	1.2141	0.2321	0.2557
INTERFERON_GAMMA_RESPONSE	0.3747	1.217	0.2706	0.2629
KRAS_SIGNALING_UP	0.3002	1.2207	0.195	0.2692
TGF_BETA_SIGNALING	0.3803	1.1928	0.2731	0.2695
ANGIOGENESIS	0.3337	1.0945	0.3071	0.3561
UNFOLDED_PROTEIN_RESPONSE	0.2909	1.0999	0.3486	0.3612
PANCREAS_BETA_CELLS	0.3087	1.0704	0.3669	0.3653
MYC_TARGETS_V2	0.3705	1.0757	0.3826	0.3692
NOTCH_SIGNALING	0.2978	1.0543	0.3597	0.3736
HEDGEHOG_SIGNALING	-0.2851	-1.0039	0.4647	0.3795
ANDROGEN_RESPONSE	0.2821	1.0294	0.4081	0.3808
EPITHELIAL_MESENCHYMAL_TRANSITION	0.2663	1.0371	0.3935	0.3825
HEME_METABOLISM	-0.3737	-1.0211	0.478	0.3945

WNT_BETA_CATENIN_SIGNALING	0.283	0.9678	0.4776	0.4457
APICAL_SURFACE	0.2599	0.9524	0.53	0.4538
INTERFERON_ALPHA_RESPONSE	0.2663	0.731	0.7094	0.7654

Table S7. Evidence from m6A2Target showing that 13 out of the 22 ferroptosis-related signatures in the FGL model were potential target gene of writers, erasers and readers (WERs) of m6A modification in leukemia cell line.

PMID	CELL LINE	WER name	Target gene	GEO accession number	High-throughput method	Perturbation direction	Log2foldchange	Perturbation effect
29290617	Mono-Mac-6	METTL14	MMP24-AS1	GSE97443	RNA-seq	knock down	1.12	up-regulated
28017614	Mono-Mac-6	FTO	PTGS2	GSE76414	MeRIP-seq	overexpression	1.07	up-regulated
29186125	MOLM13	METTL3	PTGS2	GSE94613	MeRIP-seq	knock down	-4.62	down-regula ted
29186125	MOLM13	METTL3	CHAC1	GSE94613	MeRIP-seq	knock down	3.87	up-regulated
29186125	MOLM13	METTL3	SLC7A11	GSE94613	MeRIP-seq	knock down	1.06	up-regulated
28920958	MOLM13	METTL3	SLC7A11	GSE98623	Ribo-seq	knock down	4.01	up-regulated translation
29290617	NB4	METTL14	SLC7A11	GSE97443	RNA-seq	knock down	0.62	up-regulated
29186125	MOLM13	METTL3	NOX1	GSE94613	MeRIP-seq	knock down	-1.48	down-regulated
29290617	NB4	METTL14	SLC2A6	GSE97443	RNA-seq	knock down/alternative splicing	1.91	up-regulated/ knock down
29290617	Mono-Mac-6	METTL14	SLC2A6	GSE97443	RNA-seq	knock down	1.68	up-regulated
28017614	Mono-Mac-6	FTO	SLC2A6	GSE76414	MeRIP-seq	overexpression	-1.49	down-regulated
29186125	MOLM13	METTL3	SLC2A6	GSE94613	MeRIP-seq	knock down	-1.16	down-regulated
29290617	Mono-Mac-6	METTL14	GCH1	GSE97443	RNA-seq	knock down	-1.48	down-regulated
28017614	Mono-Mac-6	FTO	GCH1	GSE76414	MeRIP-seq	overexpression	2.1	up-regulated
28017614	Mono-Mac-6	FTO	PLIN2	GSE76414	MeRIP-seq	overexpression	1.02	up-regulated
28017614	Mono-Mac-6	FTO	ENPP2	GSE76414	MeRIP-seq	overexpression	2.6	up-regulated
29290617	Mono-Mac-6	METTL14	MYB	GSE97443	RNA-seq	alternative splicing	-	knock down
29186125	MOLM13	METTL3	MYB	GSE94613	MeRIP-seq	knock down	-0.63	down-regulated
29290617	NB4	METTL14	MYB	GSE97443	RNA-seq	alternative splicing	-	knock down
29186125	MOLM13	METTL3	GPT2	GSE94613	MeRIP-seq	knock down	2.48	up-regulated
29290617	NB4	METTL14	IDH1	GSE97443	RNA-seq	knock down	0.62	up-regulated
29290617	Mono-Mac-6	METTL14	ZNF419	GSE97443	RNA-seq	alternative splicing	-	knock down

Note: WERs: writers, erasers and readers (WERs) of m6A modification.

Table S8. Comparison of FGL model and other adult AML models.

Models	FGL prognostic model	Gene mutation model	One genetic prediction model<br><br>One clinical records-based model	Immune Risk Score Model
Application	Risk stratification	Risk stratification	earlier detection, monitoring and potentially inform intervention	Risk stratification

Technique	RNA sequencing	Deep-targeted sequencing	Deep-targeted sequencing/ routinely documented clinical information	RNA sequencing
Externally validated (YES/NO)	NO	NO	YES	YES
Clinical records involved for model construction (YES/NO)	NO	NO	YES	NO
bioinformatic prediction involved for model construction (YES/NO)	NO	NO	NO	YES
Refinement to the current 2017 ELN classification (benefited group)	intermediate-risk group	low-risk group<br><br>intermediate-risk group<br><br>high-risk group	NA (both models were designed for occurrence prediction)	low-risk group
Comprehensiveness of modeling	+<br><br>Based on expression levels of 22 genes	++<br><br>Based on somatic mutation of 81 cancer- and leukemia-associated genes	++<br><br>Based on somatic mutation of 111 genes implicated in myeloid leukaemogenesis (genetic model)<br><br>Age, gender, lab features (top 50 most frequent lab tests), BMI features (clinical records-based model)	+<br><br>Based on six immune cell subsets
Reference	This study	Eisfeld et al 2020	Abelson et al 2019	Wang et al 2021
